# Microvesicles secreted by macrophages shuttle invasion-potentiating microRNAs into breast cancer cells

**DOI:** 10.1186/1476-4598-10-117

**Published:** 2011-09-22

**Authors:** Mei Yang, Jingqi Chen, Fang Su, Bin Yu, Fengxi Su, Ling Lin, Yujie Liu, Jian-Dong Huang, Erwei Song

**Affiliations:** 1 Breast Tumor Center, Sun-Yat-Sen Memorial Hospital, Sun-Yat-Sen University, Guangzhou, PR China; 2 Department of Biochemistry, University of Hong Kong, Pokfulam, Hong Kong SAR, China; 3 LIN BAI-XIN Research Center of Medicine, Sun-Yat-Sen Memorial Hospital, Sun-Yat-Sen University, Guangzhou, PR China; 4 Department of Rheumatology, The First Affiliated Hospital, Shantou University Medical College, Shantou city, Guangdong, PR China

## Abstract

**Background:**

Tumor-associated macrophages (TAMs) are alternatively activated cells induced by interleukin-4 (IL-4)-releasing CD4^+ ^T cells. TAMs promote breast cancer invasion and metastasis; however, the mechanisms underlying these interactions between macrophages and tumor cells that lead to cancer metastasis remain elusive. Previous studies have found microRNAs (miRNAs) circulating in the peripheral blood and have identified microvesicles, or exosomes, as mediators of cell-cell communication. Therefore, one alternative mechanism for the promotion of breast cancer cell invasion by TAMs may be through macrophage-secreted exosomes, which would deliver invasion-potentiating miRNAs to breast cancer cells.

**Results:**

We utilized a co-culture system with IL-4-activated macrophages and breast cancer cells to verify that miRNAs are transported from macrophages to breast cancer cells. The shuttling of fluorescently-labeled exogenous miRNAs from IL-4-activated macrophages to co-cultivated breast cancer cells without direct cell-cell contact was observed. miR-223, a miRNA specific for IL-4-activated macrophages, was detected within the exosomes released by macrophages and was significantly elevated in the co-cultivated SKBR3 and MDA-MB-231 cells. The invasiveness of the co-cultivated breast cancer cells decreased when the IL-4-activated macrophages were treated with a miR-223 antisense oligonucleotide (ASO) that would inhibit miR-223 expression. Furthermore, results from a functional assay revealed that miR-223 promoted the invasion of breast cancer cells via the Mef2c-β-catenin pathway.

**Conclusions:**

We conclude that macrophages regulate the invasiveness of breast cancer cells through exosome-mediated delivery of oncogenic miRNAs. Our data provide insight into the mechanisms underlying the metastasis-promoting interactions between macrophages and breast cancer cells.

## Background

Breast cancer is the most common malignant tumor in women, and distal metastasis of highly invasive breast cancer cells is the major cause of death in these women. Tumor-associated macrophages that infiltrate the breast cancer stroma are the most notable migratory hematopoietic cell-type in the tumor microenvironment and function to promote the invasiveness of breast cancer cells [1-4]. Macrophages are heterogeneous cells that respond differently to various stimulating signals and display numerous phenotypes [5]. Fully polarized M1, or classically activated, macrophages are stimulated by microbial agents or pro-inflammatory factors, such as lipopolysaccharides (LPS); whereas M2, or alternatively-activated, macrophages are responding to anti-inflammatory molecules, such as interleukin-4 (IL-4). The M1 and M2 macrophage phenotypes represent the two extremes of a broad range of macrophage functional states. Among these functional states, M2 macrophages activated by IL-4 have been associated with breast cancer invasion, metastasis and poor patient prognosis [2,6-10].

Previous studies have shown that TAMs promote breast cancer progression and metastasis by releasing a variety of cytokines that regulate the survival and invasiveness of tumor cells and stimulate tumor angiogenesis [11,12]. More recent data have demonstrated that macrophages are able to produce microvesicles, also known as exosomes, which shuttle proteins or microRNAs (miRNAs) into adjacent cells within the microenvironment [13,14]. Exosomes are derived from multivesicular endosomes that fuse with the plasma membrane and are shed into the extracellular space. These particles range in size from 50 to 100 nm. A wide variety of cells may release exosomes, but their contents vary depending on the cell-type of origin and its activation status [15]. One topic of considerable interest is that exosomes contain miRNAs that mediate intercellular communication [16-19]. miRNAs are short, non-coding RNAs that regulate the expression of complementary mRNAs [20]. The shuttling of these molecules between cells aids in regulating the biology of target cells. miR-223 is specific for alternatively-activated M2 macrophages induced by IL-4 and is associated with the regulation of human granulopoiesis [21].

In the present study, we demonstrate that exogenous miRNAs transfected into IL-4-activated M2 macrophages can be shuttled into co-cultivated breast cancer cells in the absence of direct cell-cell contact with the macrophages. Exosomes containing miR-223 were released by M2 cells and were then internalized by co-cultivated breast cancer cells that did not express this miRNA. The exosome-shuttled miR-223 promoted the invasiveness of breast cancer cells in vitro. This process of invasion could be inhibited by transfecting miR-223 antisense oligonucleotides (ASO) into the tumor cells. Our study provides evidence for the delivery of invasion-potentiating miR-223 by IL-4-activated macrophages to breast cancer cells via exosomes and may highlight a novel communication mechanism between TAMs and cancer cells.

## Methods

### Isolation and activation of human monocyte-derived macrophages

Institutional approval from the local research ethical committees (Internal Review and the Ethics Boards of the Sun-Yat-Sen Memorial Hospital, Sun-Yat-Sen University) was obtained prior to conducting the study. Human mononuclear cells were isolated from the peripheral blood of healthy donors by Ficoll density gradient centrifugation at 450 × *g *for 25 min at room temperature. The mononuclear cells were washed three times with PBS and plated at a density of 5 × 10^6 ^per well in 24-well plates and incubated for 1.5 h in DMEM alone. Subsequently, non-adherent cells were washed away with warm Hank's solution, and the adherent monocytes were cultured in DMEM containing 10% fetal bovine serum (FBS). Media was changed every 3 days, and the resulting monocyte-derived macrophages (MDMs) were activated by adding IL-4 (45 ng/ml) to the culture medium for 3 days.

### Cell cultures and co-cultivation

HEK-293T cells and the breast cancer cell lines, SKBR3 and MDA-MB-231, were cultured in DMEM containing 10% FBS. Co-cultivation of macrophages and breast cancer cells was performed in 24-well Boyden chambers (Corning, cat. no. 3413). Macrophages were seeded on the 0.4 μM inserts, which are permeable to supernatants but not to cellular components. Breast cancer cells were seeded in the lower chambers and grown for the indicated periods of time.

### Microarray

MicroRNA expression profiles were generated as described previously [22], and hybridization using DiscovArray miRNA arrays was performed according to the manufacturer's instructions.

### Quantitative reverse transcription-PCR

Quantitative real-time reverse transcription-PCR (qRT-PCR) for miR-223 was conducted using Real-Time PCR Universal Reagent (GenePharma Co., Ltd.) and the MX-3000P Real-Time PCR machine (Stratagene). All reactions were performed in a 20 μL reaction volume in triplicate. The primers used for miR-223 and U6 snRNA are as follows: miR-223 forward, 5'CATTGTCAGTTTGTCAAATACC3'; miR-223 reverse, 5'CCATGAGAGATCCCTAGCG3' and U6 snRNA forward, 5'ATTGGAACGATACAGAGAAGAT3'; U6 snRNA reverse, 5'GGAACGCTTCACGAATTT3'. PCR amplification consisted of an initial denaturation step at 95°C for 3 min, followed by 40 cycles of 95°C for 30 s, 62°C for 40 s, and 72°C for 30 s. Standard curves were generated, and the relative amount of miR-223 was normalized to the amount of U6 snRNA (2^- ΔCt^).

### Luciferase reporter plasmid construction

The pMIR-REPORT miRNA expression reporter (firefly luciferase reporter plasmid; Ambion) was used for plasmid construction. The 3'-UTR of *Mef2c*, containing two miR-223 target sequences, miR-223 complementary sequence (TGGGGTATTTGACAAACTGACA) and lin-4 complementary sequence (CTAGTCACACTTGAGGTCTCAGGGA), were separately cloned into the 3'-UTR of the pMIR-REPORT plasmid according to the manufacturer's instructions (Ambion).

### Invasion assay

Invasion was measured by assessing the cell migration rate through an artificial basement membrane in a modified Boyden chamber (Corning, cat. no. 3422). The membrane consisted of polycarbonate (8 μm pore diameter) and was coated on ice with Matrigel (BD Biosciences) diluted in serum-free DMEM. Cells resuspended in DMEM were seeded into the upper well of the chamber (100 μl), while the lower well was filled to the top (approximately 600 μl) with complete medium (DMEM containing 10% FBS). Cells were incubated for 4-8 h. The cells in the upper well that did not migrate were scraped off, and the cells that migrated onto the outer side of the upper well membrane were stained with crystal violet. Invading cells were observed under a microscope and counted for statistical analysis.

### Transfection of miRNA mimics and miRNA ASO

MicroRNA mimics and inhibitors (miRNA antisense oligonucleotides (ASO)) were purchased from GenePharma Co., Ltd. The sequences are provided in Table 1. In vitro transfection of miRNAs (miR-223, lin-4 and miR-NC) and miRNA ASO (miR-223 ASO and miR-NC ASO) were performed using X-tremeGENE siRNA Transfection Reagent according to the manufacturer's instructions (Roche, cat. no. 04 476 093 001).

**Table 1:** The sequences of microRNA mimics and inhibitors

hsa-miR-223	UGUCAGUUUGUCAAAUACCCCA
miR-NC (negative control)	UUCUCCGAACGUGUCACGUTT

hsa-miR-223 inhibitor (ASO)	UGGGGUAUUUGACAAACUGACA

miR-NC ASO	CAGUACUUUUGUGUAGUACAA

### Luciferase assays

To determine whether miRNAs were shuttled from macrophages to breast cancer cells, the levels of miRNAs in the target cancer cells were assessed using luciferase assays. Briefly, the pMIR-REPORT vector with either the miR-223 or the lin-4 complementary sequence in its 3'-UTR (firefly luciferase reporter vector, Ambion) were transfected into SKBR3 cells using Lipofectamine2000 (Invitrogen) according to the manufacturer's instructions. A Renilla luciferase vector (Promega) was co-transfected as an internal control for normalization. After transfection, cells were washed to remove untransfected plasmids or miRNAs. SKBR3 cells were then co-cultivated in Boyden chambers with macrophages activated with IL-4 or transfected with lin-4 mimics, as described above, for 24-72 h. pMIR-REPORT and Renilla luciferase activities were assayed using the Dual-Luciferase assay kit (Promega). pMIR-REPORT luciferase activity was normalized to the Renilla luciferase activity. To determine whether *Mef2c *mRNA is a target of miR-223, the pMIR-REPORT vector containing the 3'-UTR of *Mef2c *was co-transfected with miR-223 into HEK-293T cells. After 24-48 h of incubation, the cells were lysed, and luciferase activity was detected as described above.

### Shuttling assays for fluorescently-labeled miRNA

To further visualize the shuttling of miRNAs, Cy3-labeled miRNAs (GenePharma) were transfected into macrophages, as described above. Macrophages were washed to remove the residual transfection reagent 24-h after transfection. Macrophages carrying Cy3-miRNA were then placed onto transwell inserts, and SKBR3 cells were seeded in the lower wells of Boyden chambers. After incubation for 24-48 h, SKBR3 cells were collected for fluorescence microscopy and flow cytometric analyses.

### Western blots

Cells were lysed with RIPA lysis buffer and protease inhibitors (Sigma-Aldrich). Nuclear protein was collected according to previously described protocols [23]. A total of 20 μg of protein per sample was separated on SDS-PAGE gels and transferred onto nitrocellulose membranes. Membranes were blocked and incubated with antibodies against β-catenin (1:1000, Cell Signaling Technology, #9587) or Mef2c (1:1000, Cell Signaling Technology, #9792) overnight at 4°C. Primary antibody incubation was followed by incubation with HRP-conjugated secondary antibodies (Amersham Pharmacia). HRP signals were then visualized by enhanced chemiluminescence (Pierce).

### Confocal microscopy

Cells prepared on coverslips were fixed in 4% PFA, treated with 0.3% Triton X-100, blocked with 5% BSA and incubated with an anti-β-catenin antibody (1:200, Cell Signaling Technology, #9587) overnight at 4°C. After being washed with PBS, cells were incubated with a FITC-conjugated secondary antibody for 1 h and counterstained with PI prior to inspection under a confocal microscope.

### Immunofluorescence

SKBR3 breast cancer cells were co-cultured with Cy3-preloaded macrophages for 24-48 h. After co-culture, both macrophages and SKBR3 were fixed in 4% PFA, treated with 0.1% Triton X-100, blocked in 3% BSA and incubated with an anti-CD68 antibody (1:200, Dako) for 2 h at room temperature. After being washed, cells were incubated with an Alexa Fluor 488-conjugated secondary antibody, and then counterstained with DAPI before inspection under fluorescence microscope.

### Immunohistochemistry

All tumor samples of invasive breast cancer were obtained from female patients at the No. 2 Affiliated Hospital, Sun-Yat-Sen University. All patient samples were collected with informed consent according to the Internal Review and the Ethics Boards of the Sun-Yat-Sen Memorial Hospital, Sun-Yat-Sen University. Samples were fixed, paraffin embedded and sectioned into 5- μM slices. Macrophages were visualized by immunohistochemistry staining using an anti-CD68 (1:200, Dako) antibody. Bound primary antibody was detected by using a horseradish peroxidase conjugated secondary antibody, which was then developed in DAB solution (Dako). Pictures were taken under a light microscope.

### Exosome purification and labeling

The same number of IL-4-activated or unactivated macrophages were cultured in exosome-free medium (DMEM containing 10% FBS that was depleted of exosomes by ultra-centrifuging at 100,000 × *g *for 18 h). Conditioned media were collected after 3-5 days of incubation. Exosomes were purified by differential centrifugation. Briefly, the conditioned media were centrifuged at 500 × *g *for 30 min and 16,500 × *g *for 20 min to eliminate cells and cellular debris, respectively. Supernatants were filtered through 0.22 μm filters. Exosomes were pelleted by ultracentrifugation at 120,000 × g for 180 min, washed in PBS, pelleted again and resuspended in PBS. Exosome preparations were stained with CM-DiI (CellTracker, C7000), a fluorescent dye that labels the plasma membrane, according to the manufacturer's instructions. Next, exosomes were diluted in complete medium (DMEM with 10% FBS) and were added into the cell cultures. At the indicated time points, cells were examined under a confocal microscope and analyzed using flow cytometry.

### RNase treatment of Exosomes

The culture of unactivated and IL-4-activated macrophages and the process through which exosomes were collected are described above. Exosomes were treated with RNase according to previously described protocols [24]. Briefly, separated exosomes were incubated with RNase A at a final concentration of 100 U/ml, with or without 1% Triton X-100, at room temperature for 30 min. Exosomes were washed with PBS to remove residual RNase and Triton X-100. Exosomes were incubated with breast cancer cells prior to performing invasion assays.

### Electronic microscopy

Exosome preparations were mixed with equal quantities of freshly prepared 4% paraformaldehyde for 20 min. Samples were washed in water, pelleted by ultracentrifugation and then fixed for 5 min in 1% glutaraldehyde. After this process, exosomes were re-suspended in water, and 5 μl of the samples were loaded onto carbon-coated formvar grids. Exosomes were stained for 10 min with saturated aqueous uranyl and examined using an electron microscope (Hitachi S-4800 FEG SEM).

### Statistical analyses

All data are expressed as mean ± SD. Statistical analyses were performed using paired Student's t-tests.

## Results

### Co-cultivation with IL-4-activated macrophages elevates miR-223 levels in breast cancer cells

**Figure 1:**
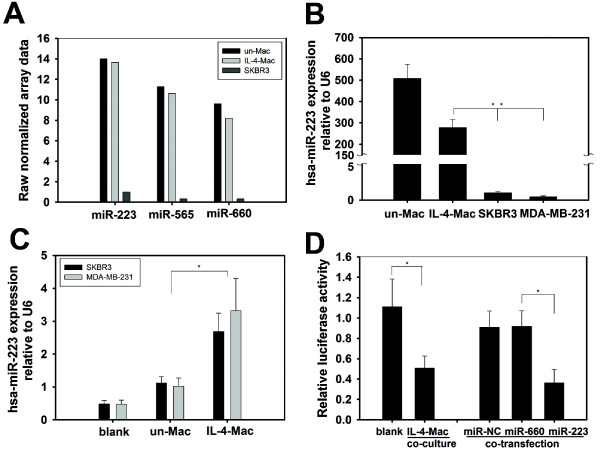
**miR-223 was up-regulated in breast cancer cell lines co-cultured with IL-4-activated macrophages**. (A) miRNA profiling in macrophages and the breast cancer cell line SKBR3 using the DiscovArray miRNA array. Three of the miRNAs that were present at high levels in macrophages but absent in SKBR3 cells are shown. (Un-Mac, unactivated macrophages; IL4-Mac, IL-4-activated macrophages). (B) Quantitative real-time PCR (qRT-PCR) confirmed a high expression level of hsa-miR-223 in macrophages but not in SKBR3 or MDA-MB-231 cells. * p < 0.05. (C) Co-culture of breast cancer cells with macrophages increased miR-223 levels in breast cancer cells. Breast cancer cell lines (SKBR3 and MDA-MB-231) were cultured alone (blank) or co-cultured with unactivated or IL-4-activated macrophages without direct cell-cell contact. miR-223 expression in SKBR3 and MDA-MB-231 cells was detected using qRT-PCR. Relative levels of miR-223 expression normalised to U6 rRNA levels are presented. * p < 0.05. (D) Functional assay of miR-223 in breast cancer cells. miR-223-targeting luciferase reporter containing a miR-223 complementary sequence within the 3'-UTR of the luciferase reporter gene was constructed. SKBR3 cells were transfected with the reporter gene and cultured alone (blank) or co-cultured with IL-4-activated macrophages. As controls, SKBR3 cells were co-transfected with the reporter gene and miR-NC, miR-660 or miR-223. Relative luciferase activities (normalised to Renilla luciferase activity) are presented. * p < 0.05.

**Figure 2:**
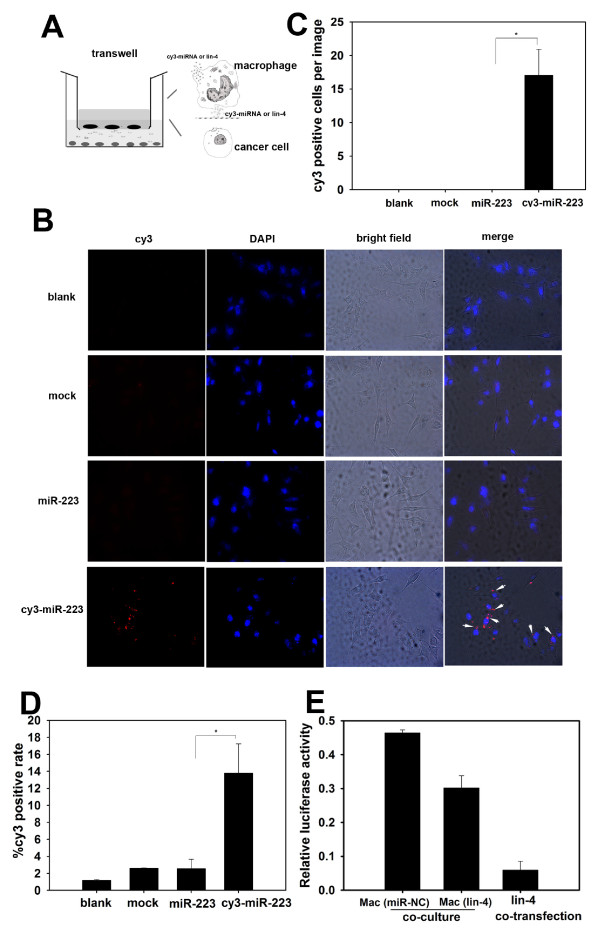
**Functional microRNA is shuttled from macrophages to breast cancer cells**. (A) Schematic illustration of a miRNA transfer model. Macrophages were preloaded with Cy3 fluorescently-labeled miR-223 or the non-mammalian miRNA lin-4. A transwell system with a 0.4- μm pore size film, which allows small size materials (e.g., miRNAs), but not cells, to pass through, was used to separate SKBR3 cells from macrophages. (B) and (C) SKBR3 cells were cultured alone (blank) or co-cultured with macrophages that were pre-transfected with reagent (mock), unlabeled miR-223 or Cy3-miR-223. The Cy3 fluorescence signal in SKBR3 cells was determined by fluorescence microscopy (B), and the percentage of Cy3-positive cells in the images was calculated in (C). Arrowheads indicate Cy3-positive SKBR3 cells, and images are shown at 200×. (D) SKBR3 cells were cultured as shown in (B), and Cy3-positive cells (%) were quantified by flow cytometry. (E) A lin-4-targeting luciferase reporter gene carrying a lin-4 complementary sequence in the 3'-UTR was transfected into SKBR3 cells. SKBR3 cells were then co-cultured in a transwell with macrophages pre-transfected with miR-NC or lin-4. SKBR3 cells that were directly transfected with lin-4 were used as a control. Luciferase activities in SKBR3 cells were measured, and relative luciferase activities (normalised to Renilla luciferase activity) are presented. * p < 0.05. (Un-Mac, unactivated macrophages; IL4-Mac, IL-4-activated macrophages).

Because TAMs located in the stroma of breast cancers are primarily M2 macrophages activated by IL-4-producing CD4^+ ^T cells [25], we mimicked this TAM-populated microenvironment by co-cultivating SKBR3 breast cancer cells with IL-4-activated MDMs in a Boyden chamber, which prevents direct cell-cell contact. To optimize a physiologically-relevant cell number ratio for the co-culture experiments, we quantified the amount of macrophage infiltration present in patient samples that were pathologically diagnosed as invasive breast cancer. As shown in Additional file 1 Figure S1(A), a large number of macrophages (CD68-positive) infiltrated breast tumors, especially in the tumor-associated stromal border, where many invasive tumor cells were also located. Because previous studies suggested that macrophages produce exosomes, which shuttle proteins or microRNAs (miRNAs) into adjacent cells within the microenvironment, we focused on the neighboring tumor cells and macrophages. We calculated the cell ratio based upon the neighboring tumor cell and macrophage populations in a local context as indicated in Additional file 1 Figure S1(B). The ratio of tumor cells to their adjacent macrophages ranged from 1:1 to 1:7. Subsequently, we tested the effects of macrophage to breast cancer cell ratios, ranging from 1:1 to 5:1, in the co-culture system. The effects of macrophages on breast cancer cells were observed at ratios starting from 1:1. First, we screened for miRNAs that were differentially expressed between macrophages and breast cancer cells using a DiscovArray miRNA microarray. All the microarray data were listed in Additional file 2 Table S1. We found three microRNAs that were abundantly expressed in macrophages but not in SKBR3 or MDA-MB-231 breast cancer cells (Figure 1A). Using qRT-PCR, we confirmed that miR-223 was overexpressed in IL-4-activated MDMs but was not highly expressed in either SKBR3 or MDA-MB-231 breast cancer cells (Figure 1B). Additionally, miR-223 is involved in cancer progression [26,27]; therefore, we focused on miR-223 expression levels in breast cancer cells after co-cultivation with IL-4-activated MDMs. We seeded breast cancer cells and macrophages in co-culture Boyden chambers, as described in Figure 2A. Interestingly, breast cancer cells co-cultured with IL-4-activated macrophages exhibited a profound increase in cellular miR-223 levels (2.7 ± 0.5- and 3.3 ± 0.98-fold in SKBR3 and MDA-MB-231 cells, respectively) relative to cells that were not co-cultured (blank) or were co-cultured with unactivated macrophages (Figure 1C). To evaluate the function of elevated miR-223 in breast cancer cells, we used a luciferase reporter gene containing a sequence complementary to miR-223 in its 3'-UTR. Co-culturing with IL-4-activated macrophages reduced luciferase reporter activity in SKBR3 breast cancer cells (Figure 1D and Additional file 3 Figure S2), which suggests that the elevated miR-223 levels observed in breast cancer cells are capable of silencing target gene expression. Furthermore, direct transfection of miR-223 mimics, but not a scrambled negative control miRNA (miR-NC), also suppressed the reporter gene activity in SKBR3 cells (Figure 1D). These data suggest that co-culturing breast cancer cells with IL-4-activated macrophages increases the level of functional miR-223 in breast cancer cells.

### miRNAs released by macrophages are shuttled into breast cancer cells

To determine whether miRNAs released by IL-4-activated macrophages are shuttled into co-cultured breast cancer cells, we transfected macrophages with either Cy3-labeled miR-223 or non-mammalian lin-4 miRNA prior to co-culture with SKBR3 cells. Co-culture was performed in a 24-well Boyden chamber with a 0.4- μm insert (Figure 2A). Fluorescence microscopy analysis indicated the presence of Cy3-miRNA in SKBR3 cells, with approximately 15 positive cells per field of view, when co-cultured with macrophages transfected with Cy3-labeled miR-223. Fluorescence was not detected in cells that were not co-cultured or that were co-cultured with macrophages transfected with unlabeled miR-223 (Figure 2B &2C). Theoretically, macrophages cannot penetrate through the 0.4- μm pore size membrane. However, to verify that the co-cultivated fluorescent tumor cells were not contaminated with macrophages, we stained these cells for CD68, a macrophage marker. As shown in Additional file 4 Figure S3, after being co-cultured with Cy3-preloaded macrophages, no CD68 staining was detected among the Cy3-positive cells. Furthermore, flow cytometric analysis confirmed that 13.8% of SKBR3 cells co-cultured with IL-4-activated macrophages that were preloaded with Cy3-labeled miR-223 were positive for Cy3-miRNA (Figure 2D). These data suggest that miRNAs within macrophages can be physically transported into adjacent cancer cells. To determine whether the miRNAs shuttled from macrophages retained their gene-silencing function in the recipient cells, we used a non-mammalian miRNA, lin-4, and its target reporter gene (luciferase reporter containing a lin-4 complementary sequence in its 3'-UTR). Prior to co-cultivation, IL-4-activated macrophages were transfected with either control (NC) or lin-4 miRNA, and SKBR3 breast cancer cells were transfected with a luciferase reporter gene with a lin-4 target sequence at its 3'-UTR. Luciferase activity was suppressed in SKBR3 cells co-cultured with macrophages transfected with lin-4, while this suppression was not observed in cells co-cultured with the control NC miRNA macrophages. Transfection of SKBR3 cells with lin-4 was used as a control to demonstrate a significant reduction in luciferase activity of the lin-4 reporter gene (Figure 2E).

### Exosomes released from IL-4-activated macrophages mediate miRNA shuttling

**Figure 3:**
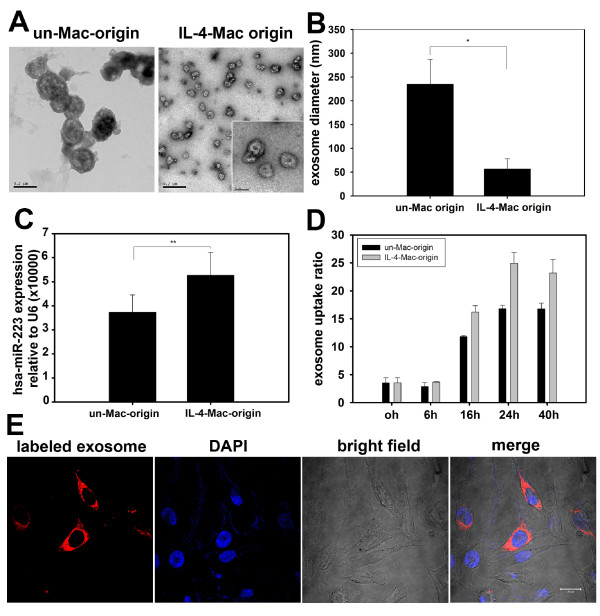
**Exosomes secreted from macrophages shuttle miR-223 to breast cancer cells**. (A) Exosomes secreted from unactivated and IL-4-activated macrophages were collected and observed with an electron microscope. Exosomes derived from unactivated macrophages (un-Mac-origin) were larger in size (100-250 nm) than those derived from IL-4-activated macrophages (IL-4-Mac-origin) (50-80 nm). Scale bars are as indicated. (B) The diameter of exosomes derived from unactivated and IL-4-activated macrophages were quantified. Data are averages of more than 10 exosomes from each group. (C) Levels of miR-223 expression in exosomes were quantified by qRT-PCR. ** p < 0.01. (D) and (E) Exosomes derived from IL-4-activated macrophages were more readily internalised by breast cancer cells. Exosomes derived from unactivated and IL-4-activated macrophages were labeled with CM-Dil and incubated with SKBR3 cells for the indicated times (0-40 h). Uptake of exosomes by cancer cells was visualized by confocal microscopy (representative images are shown in (E) and quantified by flow cytometry in (D)). (Un-Mac, unactivated macrophages; IL4-Mac, IL-4-activated macrophages).

Previous studies have demonstrated that microvesicles, or exosomes, secreted from macrophages may serve as vesicles that mediate cell-to-cell exchange of small RNAs. To further confirm that exosomes released from macrophages mediate miR-223 transfer, exosomes released from macrophages were purified by gradient centrifugation. Consistent with previous findings, electron microscopy revealed that exosomes released from the unactivated and IL-4-activated macrophages were nanometer-sized particles with bilayer membranes (Figure 3A) [16]. The exosomes derived from unactivated macrophages were much larger in size, with a diameter of 222 ± 52 nm, while those derived from IL-4-activated macrophages were smaller, with a diameter of 57 ± 21 nm (Figure 3B). Additionally, qRT-PCR data demonstrated the presence of miR-223 in the exosomes of both subtypes of macrophages, while those released from IL-4-activated macrophages contained higher levels of miR-223 than those from the unactivated cells (p < 0.01) (Figure 3C). To visualize exosome uptake in breast cancer cells, SKBR3 cells were incubated with CM-DiI-labeled exosomes that were isolated from macrophages. Internalization of CM-DiI-labeled exosomes was detected in SKBR3 cells by confocal microscopy (Figure 3E). The number of cells with internalized exosomes increased in a time-dependent manner and reached a plateau at 24 h (Figure 3D). Interestingly, exosomes released from IL-4-activated macrophages were internalized more efficiently than those released from unactivated macrophages (Figure 3D). In parallel, Cy3-labeled miR-223 from IL-4-activated macrophages was more efficiently transported into SKBR3 breast cancer cells (13.36 ± 3.52%) than the labeled miR-223 from unactivated macrophages (7.85 ± 2.84%) (Additional file 5 Figure S4). Taken together, these data suggest that macrophage-secreted exosomes mediate miR-223 shuttling.

### miR-223 promotes breast cancer cell invasion

**Figure 4:**
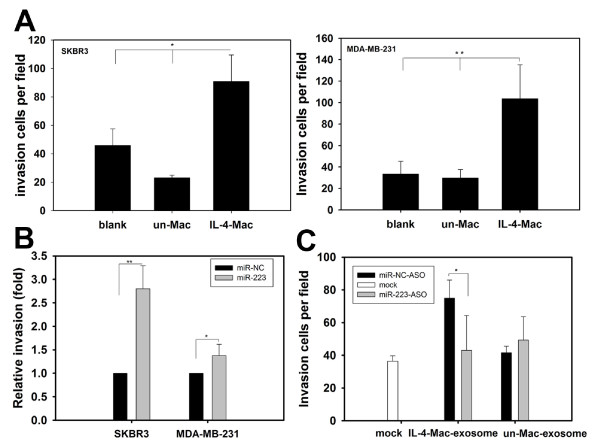
**Exosomal shuttling of miR-223 from macrophages to breast cancer cells promotes breast cancer cell invasion**. (A) IL-4-activated macrophages promote breast cancer cell invasion. The breast cancer cell lines SKBR3 and MDA-MB-231 were cultured alone (blank) or co-cultured with unactivated or IL-4-activated macrophages. Approximately 24 to 48 h after co-culture, SKBR3 and MDA-MB-231 cells were subjected to an invasion assay. Data are averages of triplicates from more than three independent experiments and are presented as the number of invading cells per field. (B) miR-223 mimics enhanced the invasion of SKBR3 and MDA-MB-231 breast cancer cells. miR-223 was transfected into SKBR3 and MDA-MB-231 cells. Cell invasion was then determined by a transwell invasion assay. Relative invasion activities are presented as fold increases in the miR-223 group. The miR-NC group was normalized to 1.0. (C) Exposure to exosomes derived from macrophages resulted in similar effects on breast cancer invasion as the actual macrophages. SKBR3 cells were incubated with exosomes derived from unactivated or IL-4-activated macrophages and then treated with miR-NC ASO or miR-223 ASO. Cell invasion assays were performed as shown in (A). Data are averages of triplicates from three independent experiments and are presented as the number of invading cells per field. * p < 0.05; ** p < 0.01. (Un-Mac-exosome, exosomes originated from unactivated macrophages; IL-4-Mac-exosome, exosomes from IL-4-activated macrophages).

miR-223 has been implicated in the progression of renal [26] and hepatocellular cancers [27]. Consistent with previous studies, co-culture with IL-4-activated macrophages enhanced breast cancer cell invasion relative to control cells cultured alone or to co-culture with unactivated macrophages (Figure 4A). To determine the biological function of miR-223 uptake by breast cancer cells, we first examined the effects of miR-223 on breast cancer cell invasion by directly transfecting miR-223 mimics into SKBR3 or MDA-MB-231 cells. Cell invasiveness was determined using a transwell invasion assay. Indeed, significantly more miR-223-transfected breast cancer cells invaded across the Matrigel-coated inserts as compared to miR-NC-transfected cells (Figure 4B). We also tested the invasion-promoting potential of exosomes from both unactivated and IL-4-activated macrophages. SKBR3 cells were incubated with exosomes purified from equal numbers of IL-4-activated and unactivated macrophages. Exosomes isolated from IL-4-activated macrophages promoted SKBR3 invasion, whereas the same effect was not observed with those isolated from unactivated macrophages (Figure 4C). Additionally, the invasion-promoting activity by exosomes derived from IL-4-activated macrophages was alleviated by miR-223-ASO (Figure 4C). Next, we treated exosomes derived from macrophages with RNase plus triton X-100 and incubated those exosomes with SKBR3 cells. Exosomes derived from IL-4-activiated macrophages treated with RNase plus triton X-100 had decreased invasion potential compared to the blank and RNase alone groups. In contrast, no significant differences were observed in the invasion potential of RNase plus triton X-100 treated exosomes derived from unactivated macrophages (Additional file 6 Figure S5). Moreover, the invasiveness of the co-cultivated breast cancer cells decreased when we treated IL-4-activated macrophages with miR-223-ASO (Additional file 7 Figure S6). Similarly, breast cancer cells preloaded with miR-223-ASO had decreased invasiveness when co-cultured with IL-4-activated macrophages (Additional file 8 Figure S7). These data suggest that miR-223 uptake by breast cancer cells is associated with the promotion of invasiveness.

### miR-223 targets the *Mef2c*-β-catenin pathway

**Figure 5:**
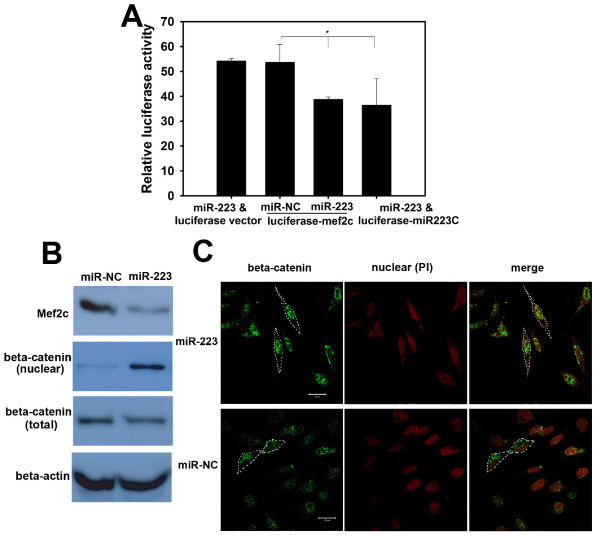
**miR-223 targets the *Mef2*c-β-catenin pathway during breast cancer cell invasion**. (A) miR-223 transfection inhibited *Mef2c *gene expression. HEK-293T cells were co-transfected with miR-223 and the *Mef2*c 3'-UTR-directed luciferase reporter (luciferase-Mef2c; *Mef2c *3'-UTR cloned into the pMIR-REPORTER). Luciferase activities were measured as described in Figure 2. Cells transfected with miR-223 and luciferase vector, miR-NC and luciferase-Mef2c, or miR-223 and luciferase-miR-223C (miR-223 complementary sequence cloned in the 3'-UTR of luciferase reporter) were used as controls. Data are averages of triplicates from three independent experiments. * p < 0.05. (B) and (C) miR-223 reduced *Mef2c *expression and resulted in nuclear accumulation of β-catenin. SKBR3 cells were transfected with miR-223 or miR-NC (control). The expression of *Mef2c *and localisation of β-catenin in breast cancer cells were then determined by cellular fractionation followed by western blot analysis with anti-*Mef2*c, β-catenin and β-actin (B). The localisation of β-catenin in SKBR3 was visualized by indirect fluorescence microscopy. Briefly, cells were incubated with an anti-β-catenin antibody followed by an incubation with a FITC-conjugated anti-mouse secondary antibody. PI was used to visualise the nuclei (C). (scale bar: 20 μm).

Previous studies have suggested that miR-223 targets the myocyte enhancer factor, *Mef2c*, in myeloid progenitor cells to inhibit their proliferation and granulocyte function [28]. Using TargetScan, we identified two miR-223 target sites in the *Mef2c *3'-UTR. To understand the mechanism by which miR-223 promotes breast cancer cell invasion, we assessed whether miR-223 targets *Mef2*c in breast cancer cells. HEK-293T cells were transfected with a luciferase reporter vector, pMIR-REPORTER-Mef2c, that contained the cloned *Mef2c *3'-UTR. After approximately 24-48 h, cells were lysed and luciferase activity was determined, as described above. As depicted in Figure 5A, transfection with miR-223 inhibited the luciferase activity of the *Mef2*c reporter gene but did not reduce the activity of the control luciferase vector that did not have the *Mef2c *3'-UTR. This inhibition was not observed in the miR-NC-transfected cells. Moreover, western blot analysis demonstrated that transfection with miR-223 reduced the levels of endogenous *Mef2c *in SKBR3 cells (Figure 5B). As *Mef2*c reduction has been linked to nuclear accumulation of β-catenin and the promotion of cell migration [29,30], we further examined whether miR-223 regulates nuclear translocation of β-catenin. Western blotting of cellular fractions demonstrated that transfection with miR-223 dramatically increased β-catenin expression in the nuclei of breast cancer cells (Figure 5B). Additionally, indirect fluorescence microscopy revealed localization of β-catenin in the nuclei of breast cancer cells transfected with miR-223 (Figure 5C). Based on these results, we conclude that miR-223 may target the *Mef2c*-β-catenin pathway to mediate breast cancer cell invasion.

## Discussion

Interactions between macrophages and breast cancer cells result in more invasive cancer cells, metastasis and poor patient prognosis. Preventing malignant breast epithelium-macrophage communication may inhibit the metastatic cascade during cancer progression [2,9], and thereby, provide important treatment targets for breast cancer therapy. Consequently, the mechanisms underlying breast cancer cell-macrophage interactions require further investigation. Most current studies focus on the cytokines, molecules or enzymes that are secreted by macrophages; however, greater attention is needed on miRNAs due to their regulatory effects on cancer cell progression [31].

Previously, miRNA profiling has been limited to tissues or cells. Evidence for circulating miRNAs is increasing, and recent studies have detected miRNAs in the peripheral blood of patients [32]. These peripheral blood-isolated miRNAs could potentially be used as biomarkers for certain diseases [33,34]. In these studies, microvesicles/exosomes were found to serve as mediators of miRNA circulation. Additional studies have suggested that exosomes can be secreted by many cells, including T cells [35], B cells [36], mast cells [16], dendritic cells [37], cancer cells [38] and macrophages [39]. Using ultracentrifugation, we isolated exosomes that were released from macrophages. We demonstrated that these macrophage-derived exosomes have classic structures consistent with previous reports [16]. Exosomes have been shown to contain protein, RNA and DNA, and much work has focused on understanding the mechanisms of exosome-mediated cell-cell interactions [40]. Some of these interactions involve communication between different cell types and convey regulatory effectors [41,42]; whereas, others occur among cells of the same type [15]. Our knowledge regarding communication between macrophages and tumor cells, however, is limited to physical contact and cytokine or chemokine secretion [43]. More recently, human macrophages were found to release exosomes containing migration-promoting enzymes [44].

Therefore, exosomes may mediate the interactions between macrophages and target cells by shuttling functional miRNAs between them. Within the breast cancer microenvironment, it is possible for macrophages to communicate with cancer cells by transferring miRNAs via exosomes. For this study, we utilized a transwell system to mimic the tumor microenvironment; this system allows macrophages to communicate with breast cancer cells without physical contact. We detected a macrophage-dominant miRNA, miR-223, that showed an increase in breast cancer cells following co-culture with macrophages. To verify miRNA transport between macrophages and breast cancer cells, we transfected Cy3-labeled miR-223 into macrophages and tracked its movement within the co-culture system. Once fluorescently-labeled miRNAs were secreted from the macrophages and captured by breast cancer cells, the fluorescent Cy3 signal could be detected in the breast cancer cells. As demonstrated by confocal microscopy and flow cytometric analyses, Cy3-positive breast cancer cells were observed, which indicates miRNA transfer. Additionally, functional miRNA delivery was confirmed using lin-4, a miRNA that is not expressed in mammalian cells.

Next, we incubated breast cancer cells with exosomes treated with RNase and triton X-100 to degrade the RNA. The depletion of RNAs in exosomes significantly decreased the invasive potential of exosomes derived from IL-4-activated macrophages. In order to determine the specific function of miR-223, we transfected miR-223-ASO into SKBR3 cells and then incubated these cells with exosomes. The invasion-promoting activity of exosomes derived from IL-4-activated macrophages on miR-223-ASO-treated SKBR3 cells was dramatically reduced. Moreover, when miR-223 expression in IL-4-activated macrophages was reduced, the invasiveness of co-cultivated breast cancer cells also decreased. Thus, it is likely that the miR-223 delivered from macrophages, and not the endogenous miR-223, promotes breast cancer cell invasion.

MDMs (monocyte-derived macrophages), prior to activation by IL-4, were not polarized and were relatively quiescent. The invasion potential of breast cancer cells co-cultured with either unactivated macrophages or exosomes released from unactivated macrophages was not different from that observed for the untreated cells. When incubated with IL-4, macrophages became activated and polarized. IL-4, the signature cytokine of a T_H_-2 type immune response, is a major activator of the TAM phenotype in the tumor microenvironment, and the effects of IL-4 collectively prime TAM with the ability to promote tumor growth, invasion and metastasis [45]. When activated by IL-4, macrophages change their cellular morphology, gene expression profile and secretory pattern [46]. TAMs selectively secrete regulators or effectors that promote tumor proliferation, invasion and metastasis [47]. miR-223 is a miRNA associated with tumor progress. Therefore, when activated by IL-4, macrophages would selectively secrete more invasion-potentiating cytokines, enzymes and miRNAs, including miR-223, that are able to regulate the characteristics of target cells. Our data demonstrate that there are differences between exosomes from unactivated and from IL-4-activated macrophages in terms of size, miR-223 content, and invasion-potential.

There is a profound impact of macrophages on breast cancer cells relative to biological function. Therefore, the identification and verification of these cellular interactions may provide additional strategies to negate the tumor promoting functions of TAMs by targeting those regulators and/or effectors which could have significant potential in anticancer therapies. In our studies, IL-4-activated macrophages efficiently secrete and deliver miR-223 to breast cancer cells, and miR-223 is responsible for macrophage-promoting breast cancer cell invasion. Clinically, based on this data, we could identify macrophage infiltration using CD68 staining, and then separate tumor cells for analysis of miR-223 expression. If greater levels of macrophage infiltration were observed in combination with up-regulation of miR-223 expression in tumor cells, which would indicate a more aggressive breast cancer phenotype according to our data, then the patient may have a poor prognosis and alternative treatments might be necessary. Inhibition of miR-223 in tumor cells and/or TAMs might be helpful. Furthermore, quantification and/or detection of miR-223 in the serum may indicate tumor aggressiveness. Therefore, it may be of great significance to designate miRNAs as diagnostic or prognostic markers for breast cancer. This will require extensive additional work to detect miR-223 in patient serum and calculate the relationship of miR-223 with prognosis, survival rate, etc.

Additionally, we explored the targets of miR-223, and a possible mechanism through which miR-223 may promote breast cancer cell invasion. Our data do not clearly demonstrate the invasion pathway utilized; however, our observations indicate that the involvement of the miR-223/Mef2c/β-catenin pathway is plausible. Additionally, a miRNA has many targets and may function through multiple different pathways. Currently, we are trying to find additional targets and verify their effects using gain- and loss-of-function assays.

The results of this study provide the first evidence suggesting that macrophages can transfer miRNA via exosomes to breast cancer cells. We found that the vesicular miRNA is responsible for macrophage-promoting breast cancer cell invasion, and we have provided a rationale for therapeutically targeting miR-223 in M2 macrophages or exosomal miR-223 from M2 macrophages.

## Conclusions

In conclusion, this study demonstrates that functional miRNAs can be transported from macrophages to breast cancer cells. Exosomes secreted from IL-4-activated macrophages shuttle miR-223 into breast cancer cells, and miR-223 promotes breast cancer cell invasion. Thus, our study provides insights into the mechanisms of cell-cell interactions through which macrophages regulate the invasiveness of breast cancer cells via the exosomal-mediated delivery of oncogenic miRNAs.

## Competing interests

The authors declare that they have no competing interests.

## Authors' contributions

MY designed and conducted the majority of the experiments, interpreted and analyzed the data, and wrote the manuscript. JQC, LL and YJL performed a subset of the breast cancer cell-invasion assays. FS contributed to the macrophage cell culture and quantitative RT-PCR assays. BY contributed to the luciferase reporter construction. FXS provided the clinical breast tumor samples and helped revise the manuscript. JDH contributed to the project design, supervised the experiments and revised the manuscript. EWS designed the project, supervised the experiments and helped draft and revise the manuscript. All authors read and approved the final manuscript.

## Supplementary Material

Additional file 1 **Figure S1. Macrophages infiltration in breast tumor**. Immunohistochemical staining of macrophage marker: CD68 in invasive breast cancer samples. (A) Macrophage distribution in invasive breast cancer of patients #1 and #2. Representative regions in #1 and #2 (200X) images were marked by rectangle and shown in (B). (B) Typical examples of tumour cells with neighbouring macrophages. In the marked regions, macrophages were those brown cells and adjacent tumour cells were marked with *. Click here for file

Additional file 2 **Table S1. Microarray data**. MicroRNA expression profiles of breast cancer cell SKBR3, unactivated and IL-4 activated macrophages were generated using DiscovArray miRNA arrays. All the microarray data were listed in the table. Click here for file

Additional file 3 **Figure S2. Luciferase assay of miR-223 activity in SKBR3 breast cancer cells**. miR-223-targeting luciferase reporter containing a miR-223 complementary sequence within the 3'-UTR of the luciferase reporter gene was constructed. SKBR3 cells were transfected with the reporter gene and cultured alone (blank) or co-cultured with IL-4-activated or unactivated macrophages. As controls, SKBR3 cells were co-transfected with the reporter gene or miR-NC. Relative luciferase activities (normalised to Renilla luciferase activity) are presented. * p < 0.05. Click here for file

Additional file 4 **Figure S3. The fluorescent Cells co-cultured with Cy3-proloaded macrophages were CD68 negative**. SKBR3 were cultured alone or co-cultured with IL-4 activated or unactivated macrophages that were pre-transfected with Cy3-miR-223. Both macrophages and SKBR3 were then stained for macrophage marker: CD68. DAPI was used to visualize nucleus. Fluorescence signals were determined by fluorescence microscopy. Arrows indicate Cy3 signal in SKBR3 cells and images are shown at 1000×. Click here for file

Additional file 5 **Figure S4. Cy3-miR-223 was more efficiently shuttled from IL-4 activated macrophages than that from unactivated macrophages to breast cancer cells**. SKBR3 cells were cultured alone (blank) or co-cultured respectively with IL-4 activated or unactivated macrophages that were pre-transfected with reagent (mock), unlabeled miR-223 or Cy3-miR-223. Cy3-positive cells (%) were quantified by flow cytometry. ** p < 0.01. (Un-Mac, unactivated macrophages; IL4-Mac, IL-4-activated macrophages). Click here for file

Additional file 6 **Figure S5. Exosomes derived from IL-4 activated macrophages treated by RNase plus triton X-100 had decreased invasion potentiate**. Exosomes secreted from IL-4 activated macrophages or unactivated macrophages were treated by RNase with or without triton X-100. SKBR3 breast cancer cells were then incubated with those exosomes. Invasion assays were performed. Data are presented as the number of invading cells per field (A) and representative images of invading cells were showed in (B). ** p < 0.01. (Un-Mac, unactivated macrophages; IL4-Mac, IL-4-activated macrophages). Click here for file

Additional file 7 **Figure S6. Inhibition of miR-223 expression in IL-4 activated macrophages decreased co-cultivated breast cancer cell invasion**. IL-4 activated macrophages were treated by miR-223-ASO, miR-NC-ASO or transfection reagent only (mock) and then co-cultured with MDA-MB-231 breast cancer cells. Breast cancer cell invasion assays were then performed. Data are presented as the number of invading cells per field (A) and representative images of invading cells were showed in (B). * p < 0.05. Click here for file

Additional file 8 **Figure S7. SKBR3 pre-treated by miR-223-ASO showed decreased cell invasion when co-cultured with IL-4 activated macrophages**. Unactivated or IL-4 activated macrophages respectively co-cultured with SKBR3 that pre-treated with miR-223-ASO (miR-223-ASO treated SKBR3) or miR-NC-ASO (miR-NC-ASO treated SKBR3). SKBR3 cell invasion was then determined by transwell invasion assay. Relative invasion activities are presented as fold changes in the miR-223-ASO group. The miR-NC-ASO group was normalized to 1.0. * p < 0.05. Click here for file

## References

[B1] Lin EY Pollard JW Tumor-associated macrophages press the angiogenic switch in breast cancer Cancer Res 2007 67 5064 5066 10.1158/0008-5472.CAN-07-0912 17545580

[B2] Leek RD Lewis CE Whitehouse R Greenall M Clarke J Harris AL Association of macrophage infiltration with angiogenesis and prognosis in invasive breast carcinoma Cancer Res 1996 56 4625 4629 8840975

[B3] Allavena P Sica A Solinas G Porta C Mantovani A The inflammatory micro-environment in tumor progression: the role of tumor-associated macrophages Crit Rev Oncol Hematol 2008 66 1 9 10.1016/j.critrevonc.2007.07.004 17913510

[B4] O'Brien J Schedin P Macrophages in breast cancer: do involution macrophages account for the poor prognosis of pregnancy-associated breast cancer? J Mammary Gland Biol Neoplasia 2009 14 145 157 10.1007/s10911-009-9118-8 19350209 PMC2693782

[B5] Martinez FO Sica A Mantovani A Locati M Macrophage activation and polarization Front Biosci 2008 13 453 461 10.2741/2692 17981560

[B6] Luo Y Zhou H Krueger J Kaplan C Lee SH Dolman C Markowitz D Wu W Liu C Reisfeld RA Xiang R Targeting tumor-associated macrophages as a novel strategy against breast cancer J Clin Invest 2006 116 2132 2141 10.1172/JCI27648 16862213 PMC1513049

[B7] Bingle L Brown NJ Lewis CE The role of tumour-associated macrophages in tumour progression: implications for new anticancer therapies J Pathol 2002 196 254 265 10.1002/path.1027 11857487

[B8] Lin EY Gouon-Evans V Nguyen AV Pollard JW The macrophage growth factor CSF-1 in mammary gland development and tumor progression J Mammary Gland Biol Neoplasia 2002 7 147 162 10.1023/A:1020399802795 12465600

[B9] Pollard JW Tumour-educated macrophages promote tumour progression and metastasis Nat Rev Cancer 2004 4 71 78 10.1038/nrc1256 14708027

[B10] Joyce JA Pollard JW Microenvironmental regulation of metastasis Nat Rev Cancer 2009 9 239 252 10.1038/nrc2618 19279573 PMC3251309

[B11] Joimel U Gest C Soria J Pritchard LL Alexandre J Laurent M Blot E Cazin L Vannier JP Varin R Stimulation of angiogenesis resulting from cooperation between macrophages and MDA-MB-231 breast cancer cells: proposed molecular mechanism and effect of tetrathiomolybdate BMC Cancer 10 375 10.1186/1471-2407-10-375 PMC2918575 20637124

[B12] Luo YP Zhou H Krueger J Kaplan C Liao D Markowitz D Liu C Chen T Chuang TH Xiang R Reisfeld RA The role of proto-oncogene Fra-1 in remodeling the tumor microenvironment in support of breast tumor cell invasion and progression Oncogene 29 662 673 10.1038/onc.2009.308 PMC3032566 19966854

[B13] Ratajczak J Wysoczynski M Hayek F Janowska-Wieczorek A Ratajczak MZ Membrane-derived microvesicles: important and underappreciated mediators of cell-to-cell communication Leukemia 2006 20 1487 1495 10.1038/sj.leu.2404296 16791265

[B14] Camussi G Deregibus MC Bruno S Cantaluppi V Biancone L Exosomes/microvesicles as a mechanism of cell-to-cell communication Kidney Int 78 838 848 10.1038/ki.2010.278 20703216

[B15] Vallhov H Gutzeit C Johansson SM Nagy N Paul M Li Q Friend S George TC Klein E Scheynius A Gabrielsson S Exosomes Containing Glycoprotein 350 Released by EBV-Transformed B Cells Selectively Target B Cells through CD21 and Block EBV Infection In Vitro J Immunol 2011 186 1 73 82 10.4049/jimmunol.1001145 21106852

[B16] Valadi H Ekstrom K Bossios A Sjostrand M Lee JJ Lotvall JO Exosome-mediated transfer of mRNAs and microRNAs is a novel mechanism of genetic exchange between cells Nat Cell Biol 2007 9 654 659 10.1038/ncb1596 17486113

[B17] Ohshima K Inoue K Fujiwara A Hatakeyama K Kanto K Watanabe Y Muramatsu K Fukuda Y Ogura S Yamaguchi K Mochizuki T Let-7 microRNA family is selectively secreted into the extracellular environment via exosomes in a metastatic gastric cancer cell line PLoS One 5 e13247 10.1371/journal.pone.0013247 PMC2951912 20949044

[B18] Gourzones C Gelin A Bombik I Klibi J Verillaud B Guigay J Lang P Temam S Schneider V Amiel C Extra-cellular release and blood diffusion of BART viral micro-RNAs produced by EBV-infected nasopharyngeal carcinoma cells Virol J 7 271 10.1186/1743-422X-7-271 PMC2974674 20950422

[B19] Luo SS Ishibashi O Ishikawa G Ishikawa T Katayama A Mishima T Takizawa T Shigihara T Goto T Izumi A Human villous trophoblasts express and secrete placenta-specific microRNAs into maternal circulation via exosomes Biol Reprod 2009 81 717 729 10.1095/biolreprod.108.075481 19494253

[B20] Ambros V The functions of animal microRNAs Nature 2004 431 350 355 10.1038/nature02871 15372042

[B21] Fazi F Rosa A Fatica A Gelmetti V De Marchis ML Nervi C Bozzoni I A minicircuitry comprised of microRNA-223 and transcription factors NFI-A and C/EBPalpha regulates human granulopoiesis Cell 2005 123 819 831 10.1016/j.cell.2005.09.023 16325577

[B22] Shingara J Keiger K Shelton J Laosinchai-Wolf W Powers P Conrad R Brown D Labourier E An optimized isolation and labeling platform for accurate microRNA expression profiling Rna 2005 11 1461 1470 10.1261/rna.2610405 16043497 PMC1370829

[B23] Liu B Wang J Chan KM Tjia WM Deng W Guan X Huang JD Li KM Chau PY Chen DJ Genomic instability in laminopathy-based premature aging Nature medicine 2005 11 780 785 10.1038/nm1266 15980864

[B24] Palanisamy V Sharma S Deshpande A Zhou H Gimzewski J Wong DT Nanostructural and transcriptomic analyses of human saliva derived exosomes PLoS One 2010 5 e8577 10.1371/journal.pone.0008577 20052414 PMC2797607

[B25] DeNardo DG Barreto JB Andreu P Vasquez L Tawfik D Kolhatkar N Coussens LM CD4(+) T cells regulate pulmonary metastasis of mammary carcinomas by enhancing protumor properties of macrophages Cancer cell 2009 16 91 102 10.1016/j.ccr.2009.06.018 19647220 PMC2778576

[B26] Gottardo F Liu CG Ferracin M Calin GA Fassan M Bassi P Sevignani C Byrne D Negrini M Pagano F Micro-RNA profiling in kidney and bladder cancers Urol Oncol 2007 25 387 392 10.1016/j.urolonc.2007.01.019 17826655

[B27] Xu J Wu C Che X Wang L Yu D Zhang T Huang L Li H Tan W Wang C Lin D Circulating MicroRNAs, miR-21, miR-122, and miR-223, in patients with hepatocellular carcinoma or chronic hepatitis Mol Carcinog 50 136 142 10.1002/mc.20712 21229610

[B28] Johnnidis JB Harris MH Wheeler RT Stehling-Sun S Lam MH Kirak O Brummelkamp TR Fleming MD Camargo FD Regulation of progenitor cell proliferation and granulocyte function by microRNA-223 Nature 2008 451 1125 1129 10.1038/nature06607 18278031

[B29] Vanpoucke G Goossens S De Craene B Gilbert B van Roy F Berx G GATA-4 and MEF2C transcription factors control the tissue-specific expression of the alphaT-catenin gene CTNNA3 Nucleic Acids Res 2004 32 4155 4165 10.1093/nar/gkh727 15302915 PMC514362

[B30] Janssens B Goossens S Staes K Gilbert B van Hengel J Colpaert C Bruyneel E Mareel M van Roy F alphaT-catenin: a novel tissue-specific beta-catenin-binding protein mediating strong cell-cell adhesion J Cell Sci 2001 114 3177 3188 11590244 10.1242/jcs.114.17.3177

[B31] Yan LX Huang XF Shao Q Huang MY Deng L Wu QL Zeng YX Shao JY MicroRNA miR-21 overexpression in human breast cancer is associated with advanced clinical stage, lymph node metastasis and patient poor prognosis Rna 2008 14 2348 2360 10.1261/rna.1034808 18812439 PMC2578865

[B32] Taylor DD Gercel-Taylor C MicroRNA signatures of tumor-derived exosomes as diagnostic biomarkers of ovarian cancer Gynecol Oncol 2008 110 13 21 10.1016/j.ygyno.2008.04.033 18589210

[B33] Rosell R Wei J Taron M Circulating MicroRNA Signatures of Tumor-Derived Exosomes for Early Diagnosis of Non-Small-Cell Lung Cancer Clin Lung Cancer 2009 10 8 9 10.3816/CLC.2009.n.001 19289365

[B34] Mitchell PS Parkin RK Kroh EM Fritz BR Wyman SK Pogosova-Agadjanyan EL Peterson A Noteboom J O'Briant KC Allen A Circulating microRNAs as stable blood-based markers for cancer detection Proc Natl Acad Sci USA 2008 105 10513 10518 10.1073/pnas.0804549105 18663219 PMC2492472

[B35] Blanchard N Lankar D Faure F Regnault A Dumont C Raposo G Hivroz C TCR activation of human T cells induces the production of exosomes bearing the TCR/CD3/zeta complex J Immunol 2002 168 3235 3241 11907077 10.4049/jimmunol.168.7.3235

[B36] Raposo G Nijman HW Stoorvogel W Liejendekker R Harding CV Melief CJ Geuze HJ B lymphocytes secrete antigen-presenting vesicles J Exp Med 1996 183 1161 1172 10.1084/jem.183.3.1161 8642258 PMC2192324

[B37] Thery C Regnault A Garin J Wolfers J Zitvogel L Ricciardi-Castagnoli P Raposo G Amigorena S Molecular characterization of dendritic cell-derived exosomes. Selective accumulation of the heat shock protein hsc73 J Cell Biol 1999 147 599 610 10.1083/jcb.147.3.599 10545503 PMC2151184

[B38] Mears R Craven RA Hanrahan S Totty N Upton C Young SL Patel P Selby PJ Banks RE Proteomic analysis of melanoma-derived exosomes by two-dimensional polyacrylamide gel electrophoresis and mass spectrometry Proteomics 2004 4 4019 4031 10.1002/pmic.200400876 15478216

[B39] O'Neill HC Quah BJ Exosomes secreted by bacterially infected macrophages are proinflammatory Sci Signal 2008 1 pe8 10.1126/stke.16pe8 18272468

[B40] Denzer K Kleijmeer MJ Heijnen HF Stoorvogel W Geuze HJ Exosome: from internal vesicle of the multivesicular body to intercellular signaling device J Cell Sci 2000 113 Pt 19 3365 3374 10984428 10.1242/jcs.113.19.3365

[B41] Huber V Filipazzi P Iero M Fais S Rivoltini L More insights into the immunosuppressive potential of tumor exosomes J Transl Med 2008 6 63 10.1186/1479-5876-6-63 18973649 PMC2590595

[B42] Xiang X Poliakov A Liu C Liu Y Deng ZB Wang J Cheng Z Shah SV Wang GJ Zhang L Induction of myeloid-derived suppressor cells by tumor exosomes Int J Cancer 2009 124 2621 2633 10.1002/ijc.24249 19235923 PMC2757307

[B43] Solinas G Marchesi F Garlanda C Mantovani A Allavena P Inflammation-mediated promotion of invasion and metastasis Cancer Metastasis Rev 29 243 248 10.1007/s10555-010-9227-2 20414701

[B44] Esser J Gehrmann U D'Alexandri FL Hidalgo-Estevez AM Wheelock CE Scheynius A Gabrielsson S Radmark O Exosomes from human macrophages and dendritic cells contain enzymes for leukotriene biosynthesis and promote granulocyte migration J Allergy Clin Immunol 126 1032 1040 1040 e1031-1034 10.1016/j.jaci.2010.06.039 20728205

[B45] Wang HW Joyce JA Alternative activation of tumor-associated macrophages by IL-4: priming for protumoral functions Cell cycle 2010 9 4824 4835 10.4161/cc.9.24.14322 21150330 PMC3047808

[B46] Loke P Nair MG Parkinson J Guiliano D Blaxter M Allen JE IL-4 dependent alternatively-activated macrophages have a distinctive in vivo gene expression phenotype BMC immunology 2002 3 7 10.1186/1471-2172-3-7 12098359 PMC117781

[B47] Cao B Guo Z Zhu Y Xu W The potential role of PDGF, IGF-1, TGF-beta expression in idiopathic pulmonary fibrosis Chinese medical journal 2000 113 776 782 11776068

