# Supplementary figures and images for: Microvesicles secreted by macrophages shuttle invasion-potentiating microRNAs into breast cancer cells
Source: Mol Cancer. 2011 Sep 22;10:117. doi: 10.1186/1476-4598-10-117 (PMC3190352; doi:10.1186/1476-4598-10-117)

**A**

200X

400X

#1

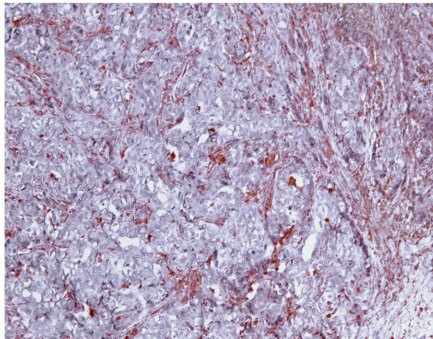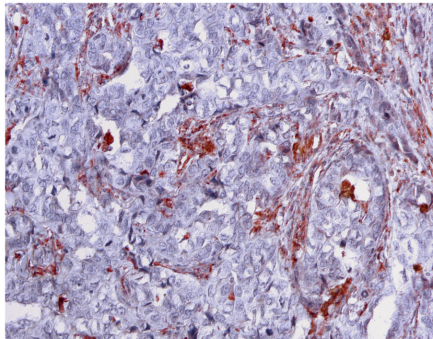

#2

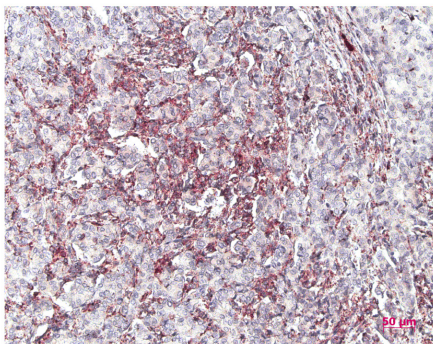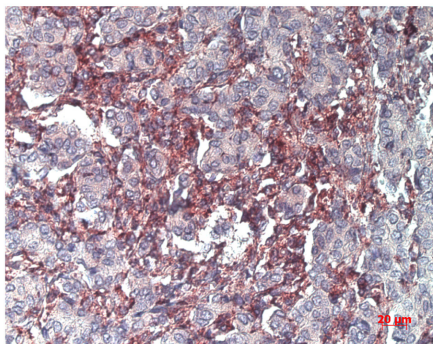**B**

#1'

#2'

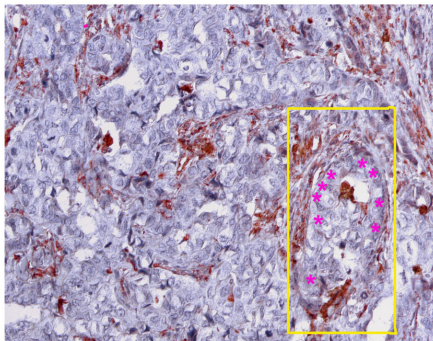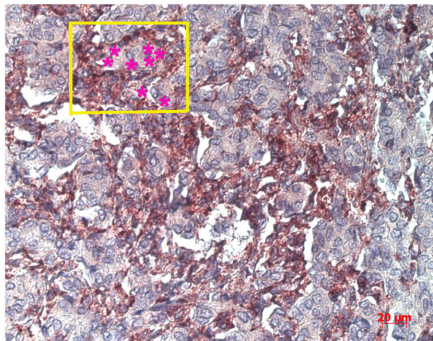

Supplement: Additional file 1 — Figure S1. Macrophages infiltration in breast tumor. Immunohistochemical staining of macrophage marker: CD68 in invasive breast cancer samples. (A) Macrophage distribution in invasive breast cancer of patients #1 and #2. Representative regions in #1 and #2 (200X) images were marked by rectangle and shown in (B). (B) Typical examples of tumour cells with neighbouring macrophages. In the marked regions, macrophages were those brown cells and adjacent tumour cells were marked with *. [file 1476-4598-10-117-S1.PDF]

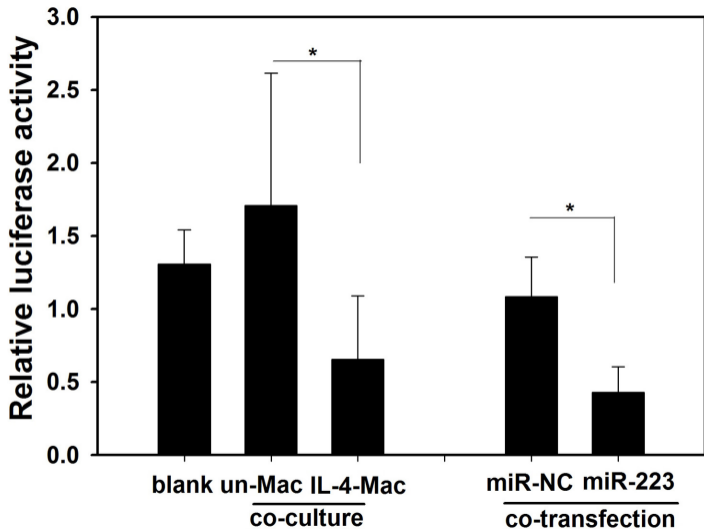

Supplement: Additional file 3 — Figure S2. Luciferase assay of miR-223 activity in SKBR3 breast cancer cells. miR-223-targeting luciferase reporter containing a miR-223 complementary sequence within the 3'-UTR of the luciferase reporter gene was constructed. SKBR3 cells were transfected with the reporter gene and cultured alone (blank) or co-cultured with IL-4-activated or unactivated macrophages. As controls, SKBR3 cells were co-transfected with the reporter gene or miR-NC. Relative luciferase activities (normalised to Renilla luciferase activity) are presented. * p < 0.05. [file 1476-4598-10-117-S3.PDF]

CD68

Cy3-miR-223

DAPI

merge

IL-4-Mac

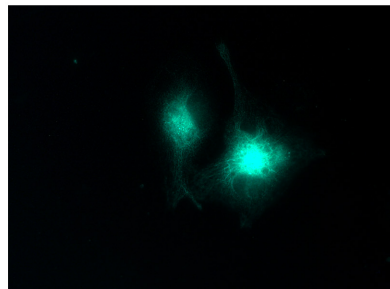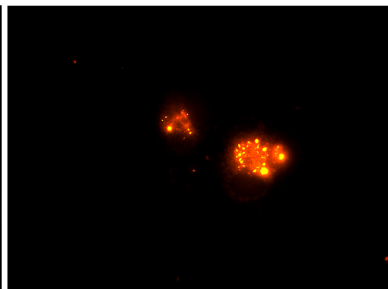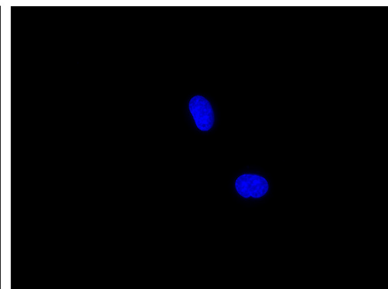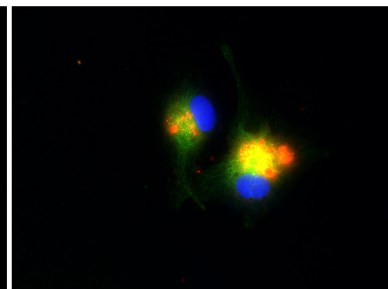

SKBR3

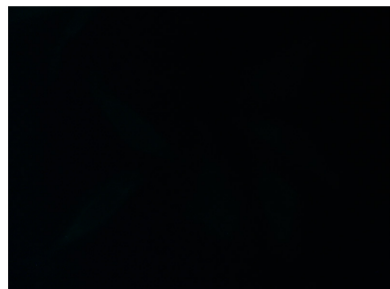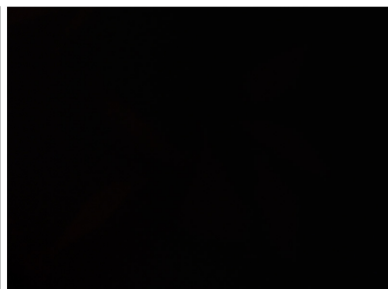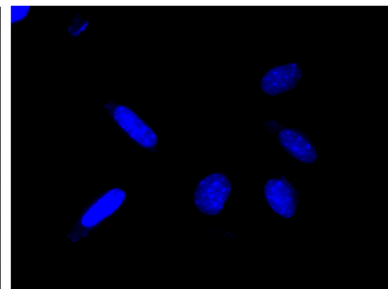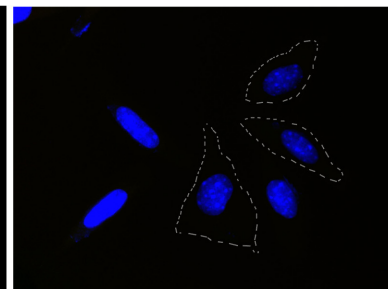SKBR3  
(IL-4-Mac)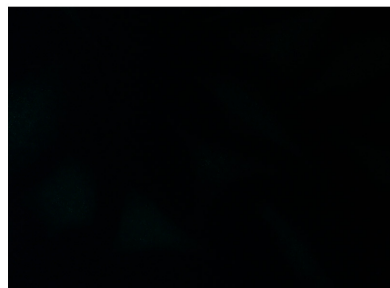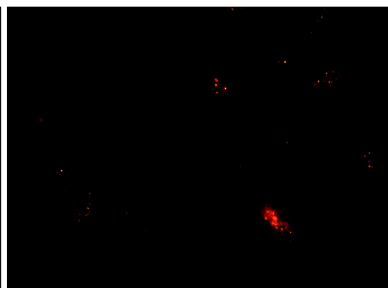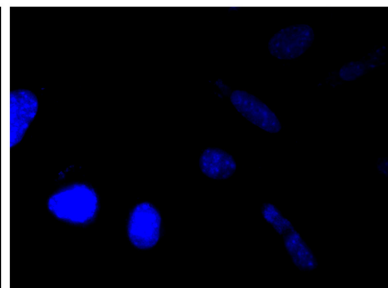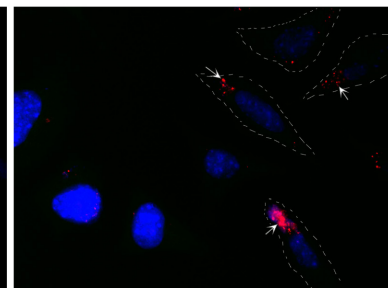SKBR3  
(un-Mac)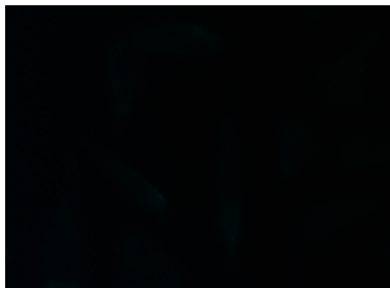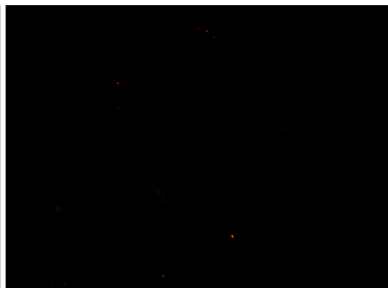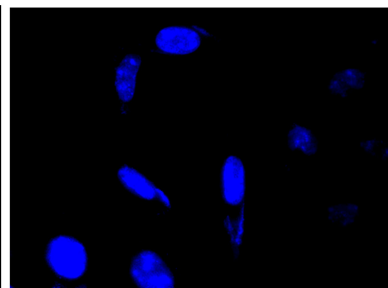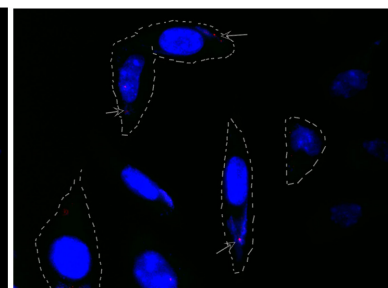

Supplement: Additional file 4 — Figure S3. The fluorescent Cells co-cultured with Cy3-proloaded macrophages were CD68 negative. SKBR3 were cultured alone or co-cultured with IL-4 activated or unactivated macrophages that were pre-transfected with Cy3-miR-223. Both macrophages and SKBR3 were then stained for macrophage marker: CD68. DAPI was used to visualize nucleus. Fluorescence signals were determined by fluorescence microscopy. Arrows indicate Cy3 signal in SKBR3 cells and images are shown at 1000×. [file 1476-4598-10-117-S4.PDF]

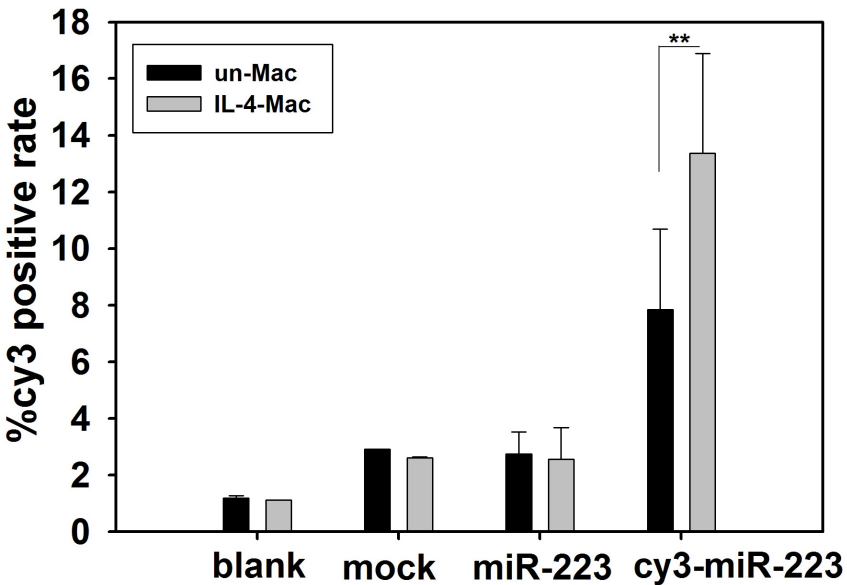

Supplement: Additional file 5 — Figure S4. Cy3-miR-223 was more efficiently shuttled from IL-4 activated macrophages than that from unactivated macrophages to breast cancer cells. SKBR3 cells were cultured alone (blank) or co-cultured respectively with IL-4 activated or unactivated macrophages that were pre-transfected with reagent (mock), unlabeled miR-223 or Cy3-miR-223. Cy3-positive cells (%) were quantified by flow cytometry. ** p < 0.01. (Un-Mac, unactivated macrophages; IL4-Mac, IL-4-activated macrophages). [file 1476-4598-10-117-S5.PDF]

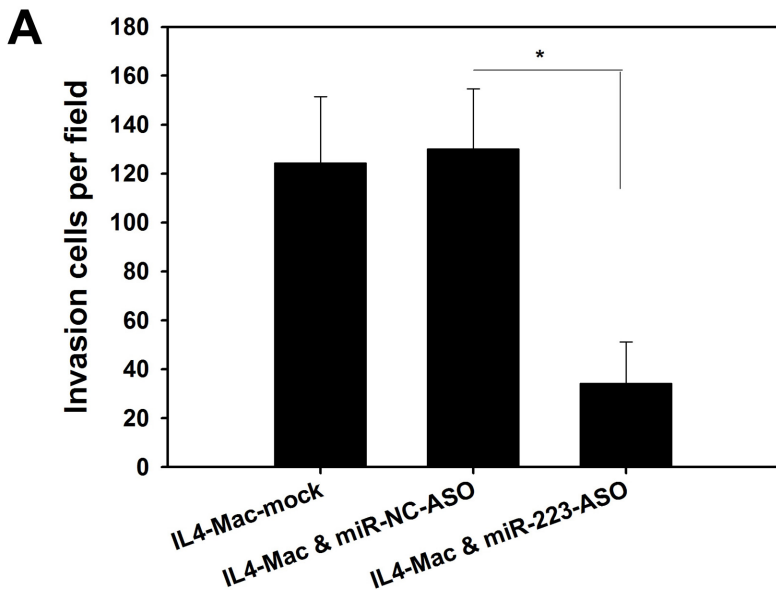

**B**

IL-4-Mac-mock

IL-4-Mac & miR-NC-ASO

IL-4-Mac & miR-223-ASO

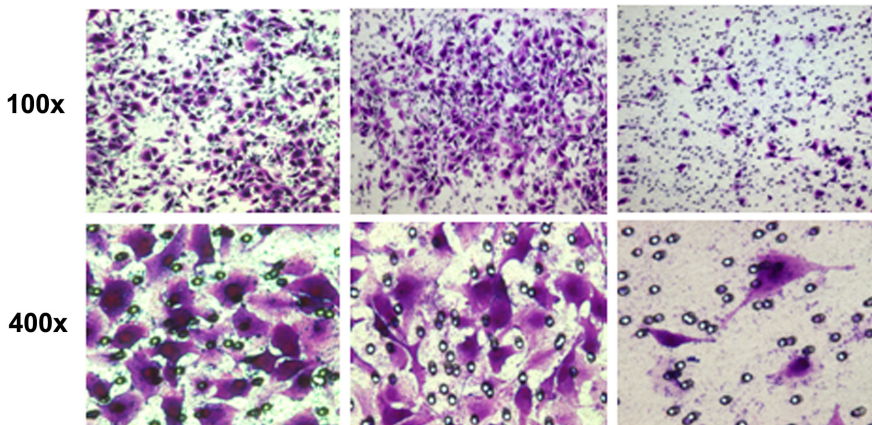

Supplement: Additional file 7 — Figure S6. Inhibition of miR-223 expression in IL-4 activated macrophages decreased co-cultivated breast cancer cell invasion. IL-4 activated macrophages were treated by miR-223-ASO, miR-NC-ASO or transfection reagent only (mock) and then co-cultured with MDA-MB-231 breast cancer cells. Breast cancer cell invasion assays were then performed. Data are presented as the number of invading cells per field (A) and representative images of invading cells were showed in (B). * p < 0.05. [file 1476-4598-10-117-S7.PDF]

Relative invasion (fold)

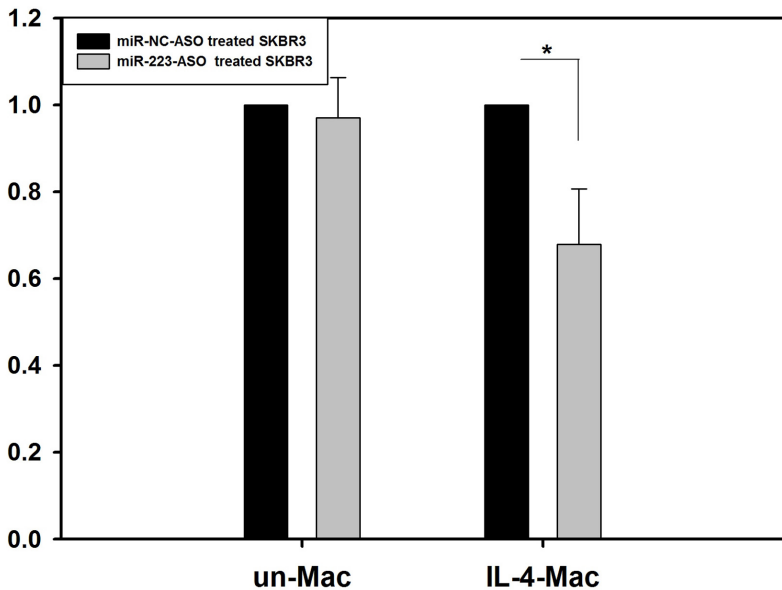

Supplement: Additional file 8 — Figure S7. SKBR3 pre-treated by miR-223-ASO showed decreased cell invasion when co-cultured with IL-4 activated macrophages. Unactivated or IL-4 activated macrophages respectively co-cultured with SKBR3 that pre-treated with miR-223-ASO (miR-223-ASO treated SKBR3) or miR-NC-ASO (miR-NC-ASO treated SKBR3). SKBR3 cell invasion was then determined by transwell invasion assay. Relative invasion activities are presented as fold changes in the miR-223-ASO group. The miR-NC-ASO group was normalized to 1.0. * p < 0.05. [file 1476-4598-10-117-S8.PDF]
