# Supplementary material for: Microvesicles secreted by macrophages shuttle invasion-potentiating microRNAs into breast cancer cells
Source: Mol Cancer. 2011 Sep 22;10:117. doi: 10.1186/1476-4598-10-117 (PMC3190352; doi:10.1186/1476-4598-10-117)
Supplement: Additional file 2 — Table S1. Microarray data. MicroRNA expression profiles of breast cancer cell SKBR3, unactivated and IL-4 activated macrophages were generated using DiscovArray miRNA arrays. All the microarray data were listed in the table. [file 1476-4598-10-117-S2.DOC]

**Table S1.** Microarray data

| CS ID | ProbeID | Annotation | **SKBR3** | **unactivated macrophage** | **IL-4 activated macrophage** |
| --- | --- | --- | --- | --- | --- |
| 1283 | 1030 | hsa-let-7a | **5.51** | **2.35** | **3.80** |
| 2323 | 1431 | hsa-let-7b | **4.46** | **2.44** | **1.69** |
| 529 | 1268 | hsa-let-7c | **9.05** | **2.06** | **2.30** |
| 13 | 2584 | hsa-let-7d | **15.28** | **14.36** | **4.69** |
| 1594 | 3217 | hsa-let-7e | **8.10** | **1.89** | **3.22** |
| 1282 | 1033 | hsa-let-7f | **4.38** | **2.06** | **2.71** |
| 1831 | 3076 | hsa-let-7f-2* | **0.51** | **0.08** | **0.06** |
| 525 | 2791 | hsa-let-7g | **4.98** | **2.60** | **3.05** |
| 1052 | 2245 | hsa-let-7i | **3.00** | **0.48** | **0.78** |
| 1794 | 1064 | hsa-miR-100 | **1.35** | **0.06** | **0.12** |
| 15 | 2562 | hsa-miR-101 | **0.19** | **0.42** | **0.30** |
| 1076 | 3015 | hsa-miR-101* | **0.44** | **0.03** | **0.06** |
| 2083 | 2984 | hsa-miR-103 | **0.59** | **0.25** | **0.44** |
| 33 | 2954 | hsa-miR-105* | **0.47** | **0.42** | **0.53** |
| 2841 | 2517 | hsa-miR-106a | **2.68** | **0.89** | **0.64** |
| 2085 | 3082 | hsa-miR-106a* | **0.49** | **0.47** | **0.03** |
| 788 | 1282 | hsa-miR-106b | **1.26** | **0.20** | **0.43** |
| 2084 | 2983 | hsa-miR-107 | **1.26** | **0.77** | **0.73** |
| 2057 | 2146 | hsa-miR-10a | **0.59** | **0.03** | **0.05** |
| 2611 | 3040 | hsa-miR-10a* | **0.59** | **0.27** | **0.20** |
| 785 | 1301 | hsa-miR-10b | **0.42** | **0.18** | **0.16** |
| 2333 | 2942 | hsa-miR-10b* | **2.14** | **0.94** | **0.72** |
| 2592 | 2943 | hsa-miR-125a-3p | **0.62** | **0.22** | **0.17** |
| 1064 | 3063 | hsa-miR-125a-5p | **1.19** | **0.10** | **0.54** |
| 292 | 2955 | hsa-miR-125b-1* | **0.51** | **0.18** | **0.20** |
| 1812 | 1377 | hsa-miR-127-3p | **0.71** | **0.17** | **0.10** |
| 1025 | 1021 | hsa-miR-129-3p | **0.36** | **0.24** | **0.36** |
| 267 | 2005 | hsa-miR-130a | **1.47** | **0.06** | **0.11** |
| 562 | 3009 | hsa-miR-130b | **2.36** | **0.99** | **0.95** |
| 546 | 2961 | hsa-miR-130b* | **0.43** | **0.20** | **0.13** |
| 1068 | 3159 | hsa-miR-135a | **0.42** | **0.21** | **0.20** |
| 811 | 3156 | hsa-miR-135a* | **1.02** | **0.48** | **0.53** |
| 809 | 3158 | hsa-miR-135b | **0.79** | **0.07** | **0.07** |
| 1328 | 3259 | hsa-miR-135b* | **0.55** | **0.22** | **0.18** |
| 547 | 2960 | hsa-miR-136 | **0.66** | **0.27** | **0.25** |
| 2349 | 3278 | hsa-miR-137 | **0.88** | **0.58** | **0.47** |
| 535 | 1878 | hsa-miR-138 | **0.57** | **0.20** | **0.20** |
| 2870 | 3141 | hsa-miR-138-1* | **0.97** | **0.44** | **0.29** |
| 2306 | 1106 | hsa-miR-140-5p | **0.57** | **0.21** | **0.17** |
| 1547 | 2117 | hsa-miR-141 | **0.93** | **0.57** | **0.53** |
| 2817 | 1169 | hsa-miR-142-5p | **0.46** | **0.43** | **0.32** |
| 17 | 1182 | hsa-miR-142-5p | **5.32** | **0.33** | **0.34** |
| 2058 | 2140 | hsa-miR-144 | **0.58** | **0.42** | **0.28** |
| 310 | 3101 | hsa-miR-144* | **1.58** | **0.77** | **0.66** |
| 309 | 3102 | hsa-miR-145* | **0.41** | **0.07** | **0.10** |
| 2067 | 1409 | hsa-miR-146a | **0.56** | **1.10** | **0.96** |
| 795 | 2237 | hsa-miR-146b-5p | **0.82** | **0.80** | **1.18** |
| 1838 | 3269 | hsa-miR-147b | **0.83** | **0.06** | **0.07** |
| 2832 | 2899 | hsa-miR-148a* | **0.57** | **0.47** | **0.24** |
| 526 | 2772 | hsa-miR-151-5p | **0.50** | **0.12** | **0.10** |
| 2074 | 2323 | hsa-miR-153 | **0.46** | **0.44** | **0.32** |
| 1538 | 1047 | hsa-miR-154* | **0.26** | **0.12** | **0.21** |
| 2093 | 3274 | hsa-miR-155 | **1.68** | **1.76** | **1.76** |
| 2088 | 3079 | hsa-miR-155* | **0.23** | **0.18** | **0.07** |
| 1042 | 1312 | hsa-miR-15a | **0.67** | **0.33** | **0.44** |
| 1041 | 1313 | hsa-miR-15b | **0.73** | **0.40** | **0.37** |
| 1319 | 3068 | hsa-miR-15b* | **0.44** | **0.04** | **0.10** |
| 269 | 2740 | hsa-miR-16 | **1.61** | **1.99** | **1.11** |
| 294 | 3053 | hsa-miR-16-1* | **0.48** | **0.15** | **0.14** |
| 549 | 3058 | hsa-miR-16-2* | **0.45** | **0.13** | **0.13** |
| 2575 | 2896 | hsa-miR-17 | **3.20** | **0.57** | **0.49** |
| 288 | 2907 | hsa-miR-181a | **0.73** | **1.00** | **0.46** |
| 287 | 2908 | hsa-miR-181b | **0.96** | **0.71** | **0.35** |
| 1026 | 1015 | hsa-miR-181c | **0.67** | **0.48** | **0.32** |
| 285 | 2910 | hsa-miR-181c* | **0.69** | **0.34** | **0.40** |
| 286 | 2909 | hsa-miR-181d | **0.69** | **0.45** | **0.21** |
| 810 | 3157 | hsa-miR-183 | **0.65** | **0.24** | **0.33** |
| 1083 | 3208 | hsa-miR-183* | **1.14** | **0.45** | **0.50** |
| 273 | 1242 | hsa-miR-184 | **2.74** | **1.67** | **1.43** |
| 311 | 3100 | hsa-miR-185 | **1.38** | **0.99** | **0.85** |
| 786 | 1292 | hsa-miR-18a | **1.08** | **0.44** | **0.47** |
| 2822 | 1850 | hsa-miR-18a* | **0.56** | **0.03** | **0.12** |
| 1803 | 2125 | hsa-miR-18b | **1.55** | **1.14** | **0.95** |
| 2322 | 1437 | hsa-miR-190 | **1.49** | **1.17** | **0.96** |
| 1851 | 3220 | hsa-miR-190b | **0.66** | **0.16** | **0.24** |
| 2609 | 3042 | hsa-miR-191 | **0.48** | **0.50** | **0.38** |
| 800 | 2915 | hsa-miR-193b | **0.09** | **0.03** | **0.05** |
| 823 | 3108 | hsa-miR-193b* | **1.26** | **0.76** | **0.88** |
| 2066 | 1416 | hsa-miR-194 | **0.47** | **0.37** | **0.28** |
| 818 | 3013 | hsa-miR-194* | **0.45** | **0.04** | **0.14** |
| 1043 | 1311 | hsa-miR-195 | **1.46** | **0.76** | **1.71** |
| 2348 | 3179 | hsa-miR-198 | **0.68** | **0.23** | **0.28** |
| 1840 | 3267 | hsa-miR-19a | **0.72** | **0.18** | **0.16** |
| 1581 | 3266 | hsa-miR-19b | **0.57** | **0.07** | **0.18** |
| 787 | 1287 | hsa-miR-200a | **0.77** | **0.37** | **0.28** |
| 1802 | 2132 | hsa-miR-200b | **0.74** | **0.44** | **0.46** |
| 1841 | 3030 | hsa-miR-200b* | **0.65** | **0.50** | **0.43** |
| 2103 | 3128 | hsa-miR-200c | **0.53** | **0.20** | **0.27** |
| 2856 | 3091 | hsa-miR-202 | **1.25** | **0.68** | **0.43** |
| 530 | 1260 | hsa-miR-203 | **0.67** | **0.52** | **0.42** |
| 7 | 1489 | hsa-miR-204 | **0.45** | **0.04** | **0.05** |
| 1331 | 3020 | hsa-miR-208b | **0.95** | **0.57** | **0.66** |
| 1592 | 3119 | hsa-miR-20a | **2.29** | **0.33** | **0.74** |
| 802 | 2965 | hsa-miR-20a* | **0.57** | **0.32** | **0.35** |
| 2317 | 2894 | hsa-miR-20b | **3.53** | **1.20** | **1.16** |
| 1300 | 1315 | hsa-miR-21 | **5.55** | **3.93** | **15.46** |
| 2076 | 2291 | hsa-miR-210 | **0.74** | **0.13** | **0.18** |
| 16 | 2560 | hsa-miR-214 | **1.61** | **1.87** | **1.23** |
| 2618 | 3233 | hsa-miR-214* | **0.58** | **0.07** | **0.24** |
| 2563 | 1122 | hsa-miR-215 | **0.81** | **0.55** | **0.43** |
| 2060 | 2136 | hsa-miR-216a | **0.46** | **0.38** | **0.31** |
| 1544 | 1775 | hsa-miR-216b | **0.55** | **0.25** | **0.27** |
| 2871 | 3140 | hsa-miR-217 | **0.39** | **0.21** | **0.17** |
| 2820 | 1143 | hsa-miR-218 | **0.47** | **0.03** | **0.05** |
| 2098 | 3033 | hsa-miR-218-1* | **0.79** | **0.03** | **0.10** |
| 2097 | 3034 | hsa-miR-218-2* | **1.25** | **0.03** | **0.32** |
| 1570 | 2977 | hsa-miR-219-2-3p | **0.65** | **0.46** | **0.50** |
| 1284 | 1022 | hsa-miR-22 | **0.30** | **0.18** | **0.77** |
| 271 | 2607 | hsa-miR-22* | **0.44** | **0.26** | **0.19** |
| 39 | 3048 | hsa-miR-220c | **0.19** | **0.21** | **0.23** |
| 2050 | 1088 | hsa-miR-221 | **0.88** | **0.47** | **0.52** |
| 35 | 2952 | hsa-miR-221* | **0.42** | **0.45** | **0.34** |
| 272 | 2594 | hsa-miR-222 | **2.09** | **1.14** | **0.78** |
| 2091 | 3176 | hsa-miR-222* | **0.36** | **0.84** | **0.06** |
| 1326 | 3261 | hsa-miR-223 | **0.99** | **14.01** | **13.66** |
| 1317 | 3070 | hsa-miR-223* | **0.36** | **0.03** | **0.06** |
| 1587 | 3024 | hsa-miR-23a | **1.38** | **0.71** | **0.84** |
| 1333 | 3118 | hsa-miR-23a* | **0.54** | **0.09** | **0.13** |
| 1588 | 3023 | hsa-miR-23b | **1.27** | **0.42** | **0.78** |
| 565 | 3106 | hsa-miR-23b* | **0.49** | **0.05** | **0.05** |
| 1539 | 1044 | hsa-miR-24 | **1.23** | **0.81** | **0.96** |
| 312 | 3099 | hsa-miR-24-2* | **0.49** | **0.26** | **0.29** |
| 2562 | 1139 | hsa-miR-25 | **0.69** | **0.04** | **0.04** |
| 2595 | 2992 | hsa-miR-25* | **0.82** | **0.26** | **0.23** |
| 2834 | 1483 | hsa-miR-26a | **1.23** | **1.31** | **0.61** |
| 1063 | 3064 | hsa-miR-26a-2* | **0.46** | **0.05** | **0.06** |
| 1578 | 3169 | hsa-miR-26b | **1.42** | **0.65** | **1.25** |
| 2095 | 3272 | hsa-miR-26b* | **0.45** | **0.05** | **0.04** |
| 2833 | 1485 | hsa-miR-27a | **2.49** | **2.18** | **0.33** |
| 2851 | 2996 | hsa-miR-27a* | **0.50** | **0.18** | **0.12** |
| 2607 | 3280 | hsa-miR-27b | **2.96** | **0.69** | **0.32** |
| 1053 | 2922 | hsa-miR-28-5p | **0.66** | **0.12** | **0.14** |
| 50 | 3001 | hsa-miR-296-3p | **23.84** | **10.25** | **9.16** |
| 275 | 1189 | hsa-miR-297 | **0.46** | **0.23** | **0.27** |
| 1825 | 2982 | hsa-miR-298 | **1.59** | **2.23** | **2.73** |
| 2827 | 2180 | hsa-miR-299-3p | **1.53** | **0.84** | **0.43** |
| 270 | 2733 | hsa-miR-29a | **1.25** | **1.03** | **0.60** |
| 14 | 2563 | hsa-miR-29a* | **0.17** | **0.41** | **0.21** |
| 42 | 3145 | hsa-miR-29b | **1.46** | **0.91** | **0.54** |
| 2343 | 3084 | hsa-miR-29b-1* | **0.75** | **0.27** | **0.21** |
| 1071 | 3256 | hsa-miR-29b-2* | **0.56** | **0.14** | **0.17** |
| 1044 | 1310 | hsa-miR-29c | **0.98** | **0.42** | **0.62** |
| 293 | 3054 | hsa-miR-301a | **1.01** | **0.61** | **0.49** |
| 552 | 3055 | hsa-miR-301b | **0.46** | **0.24** | **0.06** |
| 2104 | 3127 | hsa-miR-302a | **0.53** | **0.28** | **0.07** |
| 1316 | 2971 | hsa-miR-302a* | **0.54** | **0.23** | **0.20** |
| 1845 | 3126 | hsa-miR-302b | **0.65** | **0.25** | **0.28** |
| 1847 | 3124 | hsa-miR-302c | **0.37** | **0.09** | **0.05** |
| 2577 | 1474 | hsa-miR-302c* | **0.43** | **0.18** | **0.15** |
| 1846 | 3125 | hsa-miR-302d | **0.45** | **0.03** | **0.13** |
| 1070 | 3257 | hsa-miR-30a | **0.77** | **0.35** | **0.43** |
| 560 | 3247 | hsa-miR-30b* | **0.82** | **0.53** | **0.36** |
| 1561 | 2281 | hsa-miR-30c | **0.49** | **0.28** | **0.24** |
| 301 | 3246 | hsa-miR-30c-1* | **0.84** | **0.65** | **0.53** |
| 531 | 1251 | hsa-miR-30d | **0.99** | **0.47** | **0.57** |
| 318 | 3293 | hsa-miR-30d* | **0.47** | **0.09** | **0.12** |
| 1820 | 2282 | hsa-miR-30e | **0.83** | **0.61** | **0.50** |
| 2049 | 1092 | hsa-miR-31 | **0.32** | **0.41** | **0.40** |
| 2866 | 3045 | hsa-miR-32* | **0.41** | **0.03** | **0.17** |
| 2842 | 2516 | hsa-miR-320 | **4.30** | **1.97** | **1.28** |
| 308 | 3003 | hsa-miR-323-5p | **1.14** | **0.18** | **0.21** |
| 2051 | 1082 | hsa-miR-324-3p | **0.42** | **0.08** | **0.12** |
| 298 | 3149 | hsa-miR-325 | **0.97** | **0.59** | **0.74** |
| 2580 | 1455 | hsa-miR-328 | **0.47** | **0.09** | **0.08** |
| 1543 | 1778 | hsa-miR-329 | **0.39** | **0.13** | **0.12** |
| 542 | 2913 | hsa-miR-337-5p | **0.51** | **0.40** | **0.25** |
| 27 | 2197 | hsa-miR-338-3p | **0.59** | **0.09** | **0.20** |
| 2829 | 2902 | hsa-miR-338-5p | **0.84** | **0.45** | **0.22** |
| 1077 | 3114 | hsa-miR-33a | **0.44** | **0.05** | **0.05** |
| 2351 | 3276 | hsa-miR-340 | **1.33** | **0.80** | **0.62** |
| 2860 | 3187 | hsa-miR-340* | **0.65** | **0.05** | **0.03** |
| 314 | 3197 | hsa-miR-342-3p | **1.10** | **0.91** | **1.30** |
| 52 | 2999 | hsa-miR-342-5p | **1.24** | **0.84** | **0.67** |
| 1584 | 3263 | hsa-miR-346 | **0.44** | **0.04** | **0.05** |
| 532 | 1247 | hsa-miR-34a | **0.36** | **0.22** | **0.51** |
| 1823 | 2932 | hsa-miR-34b | **0.33** | **0.39** | **0.33** |
| 2604 | 3183 | hsa-miR-361-3p | **0.27** | **0.03** | **0.04** |
| 2350 | 3277 | hsa-miR-361-5p | **0.58** | **0.16** | **0.13** |
| 32 | 2903 | hsa-miR-362-3p | **0.46** | **0.33** | **0.18** |
| 2566 | 1822 | hsa-miR-363 | **0.57** | **0.21** | **0.12** |
| 2059 | 2138 | hsa-miR-365 | **0.05** | **0.03** | **0.03** |
| 1537 | 1052 | hsa-miR-367 | **0.58** | **0.37** | **0.32** |
| 1540 | 1038 | hsa-miR-369-3p | **1.32** | **0.81** | **0.72** |
| 2873 | 3238 | hsa-miR-370 | **1.05** | **0.60** | **0.28** |
| 2576 | 2895 | hsa-miR-372 | **0.60** | **0.39** | **0.28** |
| 1281 | 1037 | hsa-miR-373 | **0.70** | **0.16** | **0.30** |
| 2052 | 1078 | hsa-miR-373* | **0.92** | **0.36** | **0.46** |
| 2836 | 1480 | hsa-miR-374a | **0.94** | **0.70** | **0.69** |
| 2600 | 3087 | hsa-miR-374a* | **0.75** | **0.16** | **0.08** |
| 1557 | 1924 | hsa-miR-374b | **0.83** | **0.64** | **0.55** |
| 2341 | 3086 | hsa-miR-374b* | **0.61** | **0.05** | **0.13** |
| 2585 | 2342 | hsa-miR-375 | **0.42** | **0.03** | **0.03** |
| 822 | 3109 | hsa-miR-376a* | **0.55** | **0.04** | **0.06** |
| 1814 | 1934 | hsa-miR-376b | **0.98** | **0.65** | **0.48** |
| 30 | 2905 | hsa-miR-376c | **0.78** | **0.65** | **0.44** |
| 2855 | 3092 | hsa-miR-377* | **0.42** | **0.03** | **0.08** |
| 2821 | 1851 | hsa-miR-378 | **1.27** | **1.44** | **0.90** |
| 283 | 2208 | hsa-miR-378* | **0.75** | **0.05** | **0.18** |
| 815 | 3252 | hsa-miR-379 | **1.07** | **0.74** | **0.68** |
| 1066 | 3161 | hsa-miR-379* | **0.32** | **0.34** | **0.31** |
| 2828 | 2177 | hsa-miR-380 | **0.66** | **0.27** | **0.13** |
| 2571 | 2171 | hsa-miR-381 | **0.77** | **0.30** | **0.27** |
| 2311 | 1808 | hsa-miR-410 | **0.77** | **0.44** | **0.36** |
| 1329 | 3022 | hsa-miR-421 | **0.66** | **0.64** | **0.51** |
| 1060 | 2967 | hsa-miR-422a | **0.90** | **0.80** | **1.10** |
| 1595 | 3216 | hsa-miR-423-5p | **0.81** | **0.71** | **0.71** |
| 2079 | 2936 | hsa-miR-425 | **0.42** | **0.24** | **0.21** |
| 1801 | 2134 | hsa-miR-429 | **0.65** | **0.35** | **0.42** |
| 1305 | 2267 | hsa-miR-449a | **0.43** | **0.11** | **0.12** |
| 783 | 2837 | hsa-miR-450a | **0.38** | **0.22** | **0.15** |
| 799 | 2916 | hsa-miR-452 | **1.16** | **0.78** | **0.62** |
| 556 | 3151 | hsa-miR-454 | **0.67** | **0.39** | **0.27** |
| 798 | 2917 | hsa-miR-483-5p | **1.98** | **1.35** | **1.18** |
| 279 | 1863 | hsa-miR-485-5p | **0.80** | **0.74** | **0.56** |
| 2309 | 1812 | hsa-miR-487a | **1.12** | **0.60** | **0.44** |
| 2568 | 1817 | hsa-miR-487b | **0.66** | **0.49** | **0.49** |
| 63 | 3288 | hsa-miR-488 | **0.48** | **0.20** | **0.17** |
| 1082 | 3209 | hsa-miR-489 | **0.50** | **0.31** | **0.27** |
| 2599 | 3088 | hsa-miR-491-3p | **0.77** | **0.14** | **0.11** |
| 1560 | 1911 | hsa-miR-491-5p | **1.79** | **1.09** | **1.36** |
| 2081 | 2986 | hsa-miR-492 | **0.43** | **0.46** | **0.59** |
| 2331 | 2329 | hsa-miR-493* | **0.66** | **0.22** | **0.11** |
| 538 | 2230 | hsa-miR-494 | **4.83** | **4.42** | **8.56** |
| 1051 | 2248 | hsa-miR-496 | **0.41** | **0.03** | **0.06** |
| 319 | 3292 | hsa-miR-498 | **0.49** | **0.11** | **0.16** |
| 2101 | 3130 | hsa-miR-500 | **0.61** | **0.29** | **0.21** |
| 2071 | 1942 | hsa-miR-501-5p | **0.96** | **0.26** | **0.34** |
| 2843 | 2402 | hsa-miR-502-3p | **0.81** | **0.53** | **0.21** |
| 1580 | 3167 | hsa-miR-505 | **0.60** | **0.03** | **0.06** |
| 1545 | 2123 | hsa-miR-506 | **0.42** | **0.35** | **0.25** |
| 2844 | 2343 | hsa-miR-507 | **1.10** | **0.14** | **0.04** |
| 1850 | 3221 | hsa-miR-508-3p | **0.85** | **0.15** | **0.16** |
| 2853 | 3094 | hsa-miR-509-3p | **0.78** | **0.42** | **0.27** |
| 2872 | 3139 | hsa-miR-509-5p | **0.43** | **0.08** | **0.15** |
| 41 | 3146 | hsa-miR-509-5p | **0.77** | **1.00** | **0.61** |
| 548 | 2959 | hsa-miR-510 | **0.87** | **0.59** | **0.58** |
| 1583 | 3264 | hsa-miR-511 | **0.33** | **0.12** | **0.14** |
| 1301 | 1910 | hsa-miR-512-3p | **0.43** | **0.06** | **0.22** |
| 2608 | 3279 | hsa-miR-513-5p | **1.46** | **0.50** | **0.41** |
| 2354 | 3037 | hsa-miR-514 | **0.69** | **0.51** | **0.33** |
| 819 | 3012 | hsa-miR-515-3p | **0.43** | **0.03** | **0.04** |
| 64 | 3287 | hsa-miR-516a-5p | **0.19** | **0.47** | **0.31** |
| 281 | 2217 | hsa-miR-517b | **0.88** | **0.15** | **0.21** |
| 2069 | 1946 | hsa-miR-517c | **0.50** | **0.08** | **0.07** |
| 520 | 1759 | hsa-miR-518b | **0.53** | **0.07** | **0.16** |
| 313 | 3198 | hsa-miR-518c* | **0.65** | **0.03** | **0.10** |
| 2319 | 2892 | hsa-miR-519a | **0.54** | **0.33** | **0.36** |
| 517 | 1765 | hsa-miR-519c-3p | **1.04** | **0.58** | **0.58** |
| 1567 | 2928 | hsa-miR-519e | **0.57** | **0.04** | **0.03** |
| 2862 | 3285 | hsa-miR-519e* | **0.89** | **0.09** | **0.05** |
| 776 | 1771 | hsa-miR-520a-3p | **0.52** | **0.06** | **0.08** |
| 774 | 1773 | hsa-miR-520b | **0.45** | **0.08** | **0.05** |
| 2574 | 2897 | hsa-miR-520c-3p | **0.67** | **0.17** | **0.18** |
| 773 | 1774 | hsa-miR-520e | **0.85** | **0.18** | **0.15** |
| 2054 | 1802 | hsa-miR-520f | **0.55** | **0.11** | **0.22** |
| 2335 | 2940 | hsa-miR-520g | **0.53** | **0.27** | **0.23** |
| 2565 | 1824 | hsa-miR-520h | **0.49** | **0.15** | **0.19** |
| 2359 | 3132 | hsa-miR-524-5p | **0.70** | **0.42** | **0.35** |
| 58 | 3193 | hsa-miR-526a;hsa-miR-518d-5p;hsa-miR-518f*;hsa-miR-520c-5p | **0.42** | **0.31** | **0.23** |
| 569 | 3202 | hsa-miR-526b | **0.49** | **0.06** | **0.07** |
| 775 | 1772 | hsa-miR-526b* | **0.47** | **0.06** | **0.12** |
| 2346 | 3181 | hsa-miR-532-3p | **1.01** | **0.03** | **0.12** |
| 813 | 3254 | hsa-miR-541 | **0.65** | **0.43** | **0.36** |
| 1817 | 2289 | hsa-miR-542-3p | **1.42** | **0.79** | **0.76** |
| 2857 | 3190 | hsa-miR-542-5p | **0.60** | **0.19** | **0.08** |
| 2092 | 3175 | hsa-miR-545* | **0.48** | **0.15** | **0.09** |
| 12 | 1988 | hsa-miR-548b-3p | **0.24** | **0.44** | **0.05** |
| 1293 | 2878 | hsa-miR-548b-5p | **0.23** | **0.51** | **0.38** |
| 2839 | 1978 | hsa-miR-548c-3p | **0.34** | **0.37** | **0.25** |
| 1294 | 2877 | hsa-miR-548c-5p | **0.19** | **0.31** | **0.27** |
| 1552 | 2879 | hsa-miR-548d-5p | **1.08** | **1.09** | **0.85** |
| 796 | 2232 | hsa-miR-549 | **0.49** | **0.37** | **0.30** |
| 521 | 2074 | hsa-miR-551b | **0.66** | **0.08** | **0.09** |
| 2831 | 2900 | hsa-miR-551b* | **2.08** | **0.82** | **0.42** |
| 1542 | 1779 | hsa-miR-552 | **0.71** | **0.38** | **0.35** |
| 5 | 1750 | hsa-miR-553 | **0.28** | **0.41** | **0.37** |
| 1045 | 1898 | hsa-miR-555 | **0.45** | **0.32** | **0.31** |
| 1330 | 3021 | hsa-miR-556-3p | **0.43** | **0.08** | **0.15** |
| 2854 | 3093 | hsa-miR-556-5p | **0.68** | **0.14** | **0.19** |
| 2587 | 2339 | hsa-miR-557 | **0.58** | **0.04** | **0.05** |
| 1548 | 2115 | hsa-miR-559 | **0.60** | **0.41** | **0.33** |
| 2837 | 1985 | hsa-miR-561 | **0.66** | **0.48** | **0.45** |
| 518 | 1764 | hsa-miR-562 | **0.17** | **0.47** | **0.10** |
| 789 | 1884 | hsa-miR-564 | **0.44** | **0.16** | **0.36** |
| 304 | 3243 | hsa-miR-565 | **0.32** | **11.29** | **10.61** |
| 563 | 3008 | hsa-miR-567 | **0.49** | **0.04** | **0.08** |
| 2582 | 1963 | hsa-miR-568 | **0.63** | **0.42** | **0.35** |
| 536 | 1871 | hsa-miR-575 | **0.75** | **0.42** | **0.38** |
| 797 | 2918 | hsa-miR-576-3p | **1.82** | **1.09** | **1.07** |
| 265 | 2048 | hsa-miR-578 | **0.55** | **0.11** | **0.07** |
| 2075 | 2320 | hsa-miR-580 | **0.74** | **0.16** | **0.21** |
| 1848 | 3123 | hsa-miR-582-3p | **0.42** | **0.27** | **0.32** |
| 1806 | 2885 | hsa-miR-583 | **1.01** | **0.64** | **0.52** |
| 2569 | 2176 | hsa-miR-584 | **1.19** | **0.37** | **0.35** |
| 2570 | 2173 | hsa-miR-586 | **0.73** | **0.06** | **0.06** |
| 61 | 3290 | hsa-miR-588 | **0.55** | **0.43** | **0.36** |
| 2360 | 3131 | hsa-miR-590-3p | **0.49** | **0.14** | **0.19** |
| 522 | 2064 | hsa-miR-590-5p | **0.75** | **0.44** | **0.41** |
| 2330 | 2332 | hsa-miR-592 | **0.50** | **0.27** | **0.24** |
| 2312 | 1805 | hsa-miR-595 | **1.02** | **0.17** | **0.25** |
| 777 | 2088 | hsa-miR-601 | **1.29** | **0.94** | **0.90** |
| 11 | 1990 | hsa-miR-603 | **0.61** | **0.05** | **0.13** |
| 261 | 1758 | hsa-miR-606 | **0.27** | **0.37** | **0.30** |
| 1290 | 2111 | hsa-miR-607 | **0.30** | **0.23** | **0.24** |
| 51 | 3000 | hsa-miR-608 | **1.96** | **1.15** | **0.79** |
| 794 | 2239 | hsa-miR-610 | **1.09** | **0.68** | **0.68** |
| 792 | 1881 | hsa-miR-613 | **1.02** | **0.09** | **0.19** |
| 25 | 2205 | hsa-miR-615-3p | **0.44** | **0.03** | **0.04** |
| 821 | 3110 | hsa-miR-616 | **0.57** | **0.11** | **0.21** |
| 2824 | 1842 | hsa-miR-616* | **0.61** | **0.24** | **0.16** |
| 1808 | 2883 | hsa-miR-618 | **0.70** | **0.08** | **0.04** |
| 1034 | 2101 | hsa-miR-627 | **1.04** | **0.66** | **0.69** |
| 315 | 3196 | hsa-miR-628-3p | **1.57** | **1.06** | **0.84** |
| 2099 | 3032 | hsa-miR-628-5p | **0.04** | **0.86** | **0.78** |
| 557 | 3250 | hsa-miR-629 | **0.65** | **0.33** | **0.26** |
| 1302 | 1905 | hsa-miR-630 | **0.51** | **0.24** | **0.28** |
| 1541 | 1782 | hsa-miR-634 | **0.53** | **0.49** | **0.41** |
| 1058 | 2969 | hsa-miR-637 | **0.54** | **0.05** | **0.14** |
| 2852 | 2995 | hsa-miR-638 | **7.48** | **5.46** | **2.16** |
| 2053 | 1804 | hsa-miR-648 | **0.59** | **0.18** | **0.27** |
| 790 | 1883 | hsa-miR-650 | **0.69** | **0.23** | **0.30** |
| 2329 | 2335 | hsa-miR-651 | **0.61** | **0.28** | **0.16** |
| 2077 | 2938 | hsa-miR-652 | **0.32** | **0.12** | **0.22** |
| 1065 | 3162 | hsa-miR-654-3p | **0.83** | **0.27** | **0.04** |
| 2332 | 2328 | hsa-miR-659 | **0.76** | **0.21** | **0.21** |
| 2619 | 3232 | hsa-miR-661 | **0.47** | **0.03** | **0.04** |
| 1048 | 1891 | hsa-miR-663 | **2.03** | **1.37** | **2.66** |
| 2847 | 2948 | hsa-miR-665 | **0.41** | **0.44** | **0.15** |
| 1819 | 2287 | hsa-miR-668 | **0.57** | **0.07** | **0.03** |
| 2601 | 3186 | hsa-miR-671-3p | **0.40** | **0.03** | **0.08** |
| 533 | 1880 | hsa-miR-671-5p | **1.73** | **0.73** | **0.64** |
| 10 | 1991 | hsa-miR-674 | **3.87** | **4.80** | **2.86** |
| 814 | 3253 | hsa-miR-675 | **0.71** | **0.20** | **0.29** |
| 48 | 3239 | hsa-miR-7 | **0.70** | **0.89** | **0.50** |
| 2875 | 3236 | hsa-miR-744 | **2.07** | **1.00** | **0.53** |
| 2094 | 3273 | hsa-miR-744* | **0.46** | **0.07** | **0.03** |
| 2086 | 3081 | hsa-miR-760 | **0.58** | **0.07** | **0.09** |
| 1038 | 2864 | hsa-miR-765 | **2.83** | **1.61** | **1.48** |
| 781 | 2841 | hsa-miR-766 | **0.30** | **0.41** | **0.50** |
| 571 | 3200 | hsa-miR-767-3p | **0.50** | **0.04** | **0.06** |
| 2106 | 3225 | hsa-miR-767-5p | **0.27** | **0.17** | **0.06** |
| 1836 | 3171 | hsa-miR-768-3p | **0.39** | **0.32** | **0.43** |
| 62 | 3289 | hsa-miR-768-5p | **0.82** | **1.77** | **0.70** |
| 1037 | 2865 | hsa-miR-769-3p | **0.65** | **0.07** | **0.08** |
| 1039 | 2861 | hsa-miR-769-5p | **0.67** | **0.20** | **0.08** |
| 1040 | 2846 | hsa-miR-801 | **0.76** | **0.36** | **0.22** |
| 295 | 3052 | hsa-miR-802 | **0.59** | **0.11** | **0.06** |
| 1324 | 3163 | hsa-miR-871 | **0.42** | **0.41** | **0.36** |
| 1310 | 2925 | hsa-miR-872 | **0.66** | **0.35** | **0.28** |
| 553 | 3154 | hsa-miR-875-5p | **0.39** | **0.04** | **0.09** |
| 44 | 3143 | hsa-miR-877 | **2.20** | **1.45** | **1.19** |
| 2859 | 3188 | hsa-miR-877* | **0.45** | **0.09** | **0.04** |
| 2339 | 2988 | hsa-miR-885-3p | **1.14** | **0.49** | **0.52** |
| 1318 | 3069 | hsa-miR-886-3p | **1.41** | **0.03** | **0.10** |
| 2858 | 3189 | hsa-miR-886-5p | **3.80** | **0.34** | **0.07** |
| 2869 | 3142 | hsa-miR-890 | **0.74** | **0.39** | **0.29** |
| 2107 | 3224 | hsa-miR-891b | **0.49** | **0.15** | **0.18** |
| 1059 | 2968 | hsa-miR-892b | **1.33** | **0.72** | **0.82** |
| 827 | 3204 | hsa-miR-9 | **0.55** | **0.12** | **0.16** |
| 1332 | 3019 | hsa-miR-9* | **0.47** | **0.46** | **0.19** |
| 567 | 3104 | hsa-miR-920 | **1.12** | **0.91** | **0.96** |
| 56 | 3095 | hsa-miR-922 | **0.92** | **0.89** | **0.51** |
| 1833 | 3174 | hsa-miR-923 | **14.46** | **8.95** | **12.45** |
| 1796 | 1054 | hsa-miR-92a | **1.35** | **0.35** | **0.62** |
| 305 | 3006 | hsa-miR-92a-1* | **0.56** | **0.30** | **0.27** |
| 2826 | 2182 | hsa-miR-92b | **0.93** | **0.10** | **0.06** |
| 2594 | 2993 | hsa-miR-92b* | **3.61** | **2.72** | **3.21** |
| 2612 | 3039 | hsa-miR-93 | **2.10** | **0.38** | **0.22** |
| 1839 | 3268 | hsa-miR-933 | **1.00** | **1.57** | **2.50** |
| 2591 | 2944 | hsa-miR-936 | **0.75** | **0.32** | **0.36** |
| 558 | 3249 | hsa-miR-939 | **2.53** | **0.92** | **0.86** |
| 570 | 3201 | hsa-miR-942 | **0.71** | **0.03** | **0.06** |
| 2830 | 2901 | hsa-miR-944 | **1.35** | **0.80** | **0.55** |
| 2835 | 1482 | hsa-miR-95 | **0.75** | **0.26** | **0.30** |
| 574 | 3297 | hsa-miR-96 | **0.48** | **0.04** | **0.07** |
| 1821 | 2934 | hsa-miR-96* | **0.41** | **0.08** | **0.07** |
| 2065 | 1423 | hsa-miR-98 | **4.85** | **2.86** | **2.53** |
| 1055 | 2920 | hsa-miR-99a* | **0.45** | **0.06** | **0.03** |
| 1795 | 1063 | hsa-miR-99b | **0.43** | **0.18** | **0.16** |
| 1054 | 2921 | hsa-miR-99b* | **0.74** | **0.03** | **0.03** |
| 830 | ?3301 | IVGN-novel-miR_3301 | **1.48** | **0.42** | **0.42** |
| 829 | ?3302 | IVGN-novel-miR_3302 | **1.10** | **0.42** | **0.91** |
| 1088 | ?3303 | IVGN-novel-miR_3303 | **2.89** | **1.31** | **1.22** |
| 1087 | ?3304 | IVGN-novel-miR_3304 | **3.41** | **1.58** | **1.44** |
| 1086 | ?3305 | IVGN-novel-miR_3305 | **0.40** | **0.30** | **0.26** |
| 1085 | ?3306 | IVGN-novel-miR_3306 | **0.65** | **0.53** | **0.48** |
| 1344 | ?3307 | IVGN-novel-miR_3307 | **0.86** | **0.37** | **0.13** |
| 1343 | ?3308 | IVGN-novel-miR_3308 | **1.84** | **1.12** | **1.00** |
| 1341 | ?3310 | IVGN-novel-miR_3310 | **0.52** | **0.31** | **0.46** |
| 1600 | ?3311 | IVGN-novel-miR_3311 | **4.78** | **1.56** | **2.48** |
| 1599 | ?3312 | IVGN-novel-miR_3312 | **0.65** | **0.40** | **0.31** |
| 1598 | ?3313 | IVGN-novel-miR_3313 | **77.17** | **25.49** | **57.59** |
| 1597 | ?3314 | IVGN-novel-miR_3314 | **89.00** | **38.82** | **75.39** |
| 1856 | ?3315 | IVGN-novel-miR_3315 | **0.55** | **0.33** | **0.25** |
| 1855 | ?3316 | IVGN-novel-miR_3316 | **0.82** | **0.47** | **0.28** |
| 1854 | ?3317 | IVGN-novel-miR_3317 | **1.55** | **1.83** | **1.82** |
| 1853 | ?3318 | IVGN-novel-miR_3318 | **8.96** | **2.80** | **4.64** |
| 2112 | ?3319 | IVGN-novel-miR_3319 | **16.87** | **10.34** | **14.22** |
| 2111 | ?3320 | IVGN-novel-miR_3320 | **2.18** | **1.36** | **1.29** |
| 2109 | ?3322 | IVGN-novel-miR_3322 | **1.63** | **1.08** | **1.25** |
| 2368 | ?3323 | IVGN-novel-miR_3323 | **1.23** | **0.80** | **0.50** |
| 2367 | ?3324 | IVGN-novel-miR_3324 | **1.02** | **0.48** | **0.37** |
| 2366 | ?3325 | IVGN-novel-miR_3325 | **0.66** | **0.28** | **0.21** |
| 2365 | ?3326 | IVGN-novel-miR_3326 | **4.04** | **0.81** | **0.67** |
| 2624 | ?3327 | IVGN-novel-miR_3327 | **0.43** | **0.77** | **0.65** |
| 2623 | ?3328 | IVGN-novel-miR_3328 | **2.96** | **0.55** | **0.38** |
| 2622 | ?3329 | IVGN-novel-miR_3329 | **1.68** | **0.53** | **0.28** |
| 2621 | ?3330 | IVGN-novel-miR_3330 | **22.87** | **9.40** | **5.80** |
| 2880 | ?3331 | IVGN-novel-miR_3331 | **0.71** | **0.44** | **0.37** |
| 2879 | ?3332 | IVGN-novel-miR_3332 | **226.44** | **78.91** | **79.27** |
| 2878 | ?3333 | IVGN-novel-miR_3333 | **0.48** | **0.10** | **0.06** |
| 2877 | ?3334 | IVGN-novel-miR_3334 | **0.39** | **0.24** | **0.22** |
| 68 | ?3335 | IVGN-novel-miR_3335 | **1.01** | **0.19** | **0.18** |
| 66 | 3337 | IVGN-novel-miR_3337 | **14.24** | **7.33** | **9.18** |
| 65 | 3338 | IVGN-novel-miR_3338 | **7.73** | **3.18** | **2.11** |
| 322 | ?3341 | IVGN-novel-miR_3341 | **5.23** | **0.90** | **1.20** |
| 321 | ?3342 | IVGN-novel-miR_3342 | **3.80** | **1.76** | **1.49** |
| 580 | ?3343 | IVGN-novel-miR_3343 | **0.86** | **1.18** | **1.45** |
| 578 | ?3345 | IVGN-novel-miR_3345 | **3.39** | **0.96** | **0.79** |
| 577 | ?3346 | IVGN-novel-miR_3346 | **0.39** | **0.35** | **0.34** |
| 836 | ?3347 | IVGN-novel-miR_3347 | **9.53** | **4.80** | **6.49** |
| 834 | ?3349 | IVGN-novel-miR_3349 | **0.51** | **0.72** | **0.95** |
| 1092 | ?3351 | IVGN-novel-miR_3351 | **2.43** | **2.13** | **2.76** |
| 1090 | ?3353 | IVGN-novel-miR_3353 | **1.56** | **0.46** | **0.57** |
| 1089 | ?3354 | IVGN-novel-miR_3354 | **22.13** | **17.46** | **25.23** |
| 1348 | ?3355 | IVGN-novel-miR_3355 | **52.40** | **52.76** | **57.53** |
| 1346 | ?3357 | IVGN-novel-miR_3357 | **2.55** | **1.85** | **4.98** |
| 1345 | ?3358 | IVGN-novel-miR_3358 | **0.56** | **0.86** | **1.15** |
| 1604 | ?3359 | IVGN-novel-miR_3359 | **0.24** | **0.61** | **1.09** |
| 1602 | ?3361 | IVGN-novel-miR_3361 | **3.20** | **2.45** | **2.63** |
| 1601 | ?3362 | IVGN-novel-miR_3362 | **0.68** | **1.09** | **1.25** |
| 1860 | ?3363 | IVGN-novel-miR_3363 | **0.25** | **0.49** | **0.72** |
| 1859 | ?3364 | IVGN-novel-miR_3364 | **0.10** | **0.45** | **0.35** |
| 1858 | ?3365 | IVGN-novel-miR_3365 | **0.92** | **1.17** | **1.05** |
| 2116 | ?3367 | IVGN-novel-miR_3367 | **0.12** | **0.33** | **0.48** |
| 2114 | ?3369 | IVGN-novel-miR_3369 | **0.79** | **0.86** | **0.77** |
| 2113 | ?3370 | IVGN-novel-miR_3370 | **1.10** | **1.21** | **1.01** |
| 2371 | ?3372 | IVGN-novel-miR_3372 | **1.72** | **1.47** | **1.23** |
| 2369 | ?3374 | IVGN-novel-miR_3374 | **1.97** | **1.69** | **1.14** |
| 2626 | ?3377 | IVGN-novel-miR_3377 | **1.16** | **0.99** | **0.46** |
| 2625 | ?3378 | IVGN-novel-miR_3378 | **2.37** | **2.37** | **1.39** |
| 2883 | ?3380 | IVGN-novel-miR_3380 | **0.72** | **0.50** | **0.42** |
| 2881 | ?3382 | IVGN-novel-miR_3382 | **1.41** | **1.76** | **0.73** |
| 84 | ?3383 | IVGN-novel-miR_3383 | **2.37** | **1.60** | **1.68** |
| 83 | ?3384 | IVGN-novel-miR_3384 | **2.31** | **0.99** | **0.85** |
| 82 | ?3385 | IVGN-novel-miR_3385 | **3.43** | **1.36** | **1.36** |
| 81 | ?3386 | IVGN-novel-miR_3386 | **4.06** | **1.78** | **1.55** |
| 340 | ?3387 | IVGN-novel-miR_3387 | **20.65** | **4.67** | **8.56** |
| 339 | ?3388 | IVGN-novel-miR_3388 | **3.60** | **1.25** | **1.43** |
| 594 | ?3393 | IVGN-novel-miR_339 | **1.11** | **0.79** | **0.80** |
| 337 | ?3390 | IVGN-novel-miR_3390 | **54.93** | **14.15** | **19.59** |
| 596 | ?3391 | IVGN-novel-miR_3391 | **0.20** | **0.17** | **0.52** |
| 595 | ?3392 | IVGN-novel-miR_3392 | **1.82** | **1.24** | **1.19** |
| 593 | ?3394 | IVGN-novel-miR_3394 | **0.75** | **0.55** | **0.40** |
| 852 | ?3395 | IVGN-novel-miR_3395 | **6.04** | **1.97** | **2.91** |
| 851 | ?3396 | IVGN-novel-miR_3396 | **13.46** | **7.22** | **15.55** |
| 850 | ?3397 | IVGN-novel-miR_3397 | **12.68** | **3.02** | **8.13** |
| 1108 | ?3399 | IVGN-novel-miR_3399 | **13.00** | **6.92** | **13.86** |
| 1106 | ?3401 | IVGN-novel-miR_3401 | **0.61** | **0.82** | **0.70** |
| 1105 | ?3402 | IVGN-novel-miR_3402 | **6.40** | **9.59** | **7.04** |
| 1364 | ?3403 | IVGN-novel-miR_3403 | **7.94** | **14.49** | **9.66** |
| 1363 | ?3404 | IVGN-novel-miR_3404 | **0.76** | **0.26** | **0.56** |
| 1361 | ?3406 | IVGN-novel-miR_3406 | **0.43** | **0.34** | **0.31** |
| 1620 | ?3407 | IVGN-novel-miR_3407 | **0.67** | **0.61** | **0.88** |
| 1619 | ?3408 | IVGN-novel-miR_3408 | **15.94** | **5.83** | **7.84** |
| 1618 | ?3409 | IVGN-novel-miR_3409 | **12.59** | **5.45** | **6.66** |
| 1617 | ?3410 | IVGN-novel-miR_3410 | **170.84** | **174.58** | **212.82** |
| 1876 | ?3411 | IVGN-novel-miR_3411 | **12.40** | **3.65** | **5.44** |
| 1875 | ?3412 | IVGN-novel-miR_3412 | **0.70** | **0.52** | **0.51** |
| 1874 | ?3413 | IVGN-novel-miR_3413 | **14.15** | **7.69** | **15.45** |
| 1873 | ?3414 | IVGN-novel-miR_3414 | **40.72** | **19.18** | **27.06** |
| 2132 | ?3415 | IVGN-novel-miR_3415 | **15.89** | **20.93** | **22.84** |
| 2131 | ?3416 | IVGN-novel-miR_3416 | **5.56** | **2.04** | **1.93** |
| 2130 | ?3417 | IVGN-novel-miR_3417 | **23.43** | **36.25** | **35.77** |
| 2129 | ?3418 | IVGN-novel-miR_3418 | **1.44** | **0.87** | **0.71** |
| 2388 | ?3419 | IVGN-novel-miR_3419 | **12.19** | **6.46** | **5.44** |
| 2387 | ?3420 | IVGN-novel-miR_3420 | **3.10** | **1.84** | **1.47** |
| 2386 | ?3421 | IVGN-novel-miR_3421 | **6.13** | **5.31** | **3.59** |
| 2385 | ?3422 | IVGN-novel-miR_3422 | **13.09** | **10.29** | **5.50** |
| 2644 | ?3423 | IVGN-novel-miR_3423 | **10.82** | **11.95** | **2.05** |
| 2643 | ?3424 | IVGN-novel-miR_3424 | **15.85** | **28.73** | **7.94** |
| 2642 | ?3425 | IVGN-novel-miR_3425 | **34.90** | **21.14** | **10.12** |
| 2641 | ?3426 | IVGN-novel-miR_3426 | **15.15** | **18.98** | **7.42** |
| 2900 | ?3427 | IVGN-novel-miR_3427 | **1.92** | **1.09** | **0.20** |
| 2899 | ?3428 | IVGN-novel-miR_3428 | **8.73** | **19.98** | **9.97** |
| 2898 | ?3429 | IVGN-novel-miR_3429 | **2.28** | **1.71** | **0.95** |
| 2897 | ?3430 | IVGN-novel-miR_3430 | **0.45** | **0.34** | **0.32** |
| 72 | ?3431 | IVGN-novel-miR_3431 | **0.47** | **0.50** | **0.45** |
| 71 | ?3432 | IVGN-novel-miR_3432 | **3.76** | **1.56** | **3.43** |
| 70 | ?3433 | IVGN-novel-miR_3433 | **19.72** | **10.23** | **12.23** |
| 69 | ?3434 | IVGN-novel-miR_3434 | **4.75** | **1.39** | **1.86** |
| 328 | ?3435 | IVGN-novel-miR_3435 | **6.99** | **1.90** | **2.51** |
| 327 | ?3436 | IVGN-novel-miR_3436 | **9.38** | **14.05** | **13.83** |
| 326 | ?3437 | IVGN-novel-miR_3437 | **11.64** | **9.36** | **8.28** |
| 584 | ?3439 | IVGN-novel-miR_3439 | **1.02** | **0.19** | **0.56** |
| 583 | ?3440 | IVGN-novel-miR_3440 | **1.16** | **0.71** | **0.70** |
| 582 | ?3441 | IVGN-novel-miR_3441 | **2.54** | **0.94** | **1.05** |
| 581 | ?3442 | IVGN-novel-miR_3442 | **0.68** | **0.21** | **0.46** |
| 840 | ?3443 | IVGN-novel-miR_3443 | **1.56** | **0.97** | **0.95** |
| 839 | ?3444 | IVGN-novel-miR_3444 | **1.12** | **0.58** | **0.59** |
| 838 | ?3445 | IVGN-novel-miR_3445 | **2.18** | **1.01** | **2.44** |
| 837 | ?3446 | IVGN-novel-miR_3446 | **0.42** | **0.13** | **0.16** |
| 1096 | ?3447 | IVGN-novel-miR_3447 | **0.35** | **0.19** | **0.30** |
| 1095 | ?3448 | IVGN-novel-miR_3448 | **0.56** | **0.49** | **0.73** |
| 1094 | ?3449 | IVGN-novel-miR_3449 | **0.60** | **0.72** | **1.01** |
| 1093 | ?3450 | IVGN-novel-miR_3450 | **0.44** | **0.17** | **0.31** |
| 1351 | ?3452 | IVGN-novel-miR_3452 | **0.75** | **0.40** | **0.36** |
| 1350 | ?3453 | IVGN-novel-miR_3453 | **0.60** | **0.16** | **0.32** |
| 1349 | ?3454 | IVGN-novel-miR_3454 | **4.50** | **4.17** | **12.20** |
| 1607 | ?3456 | IVGN-novel-miR_3456 | **0.91** | **0.09** | **0.11** |
| 1606 | ?3457 | IVGN-novel-miR_3457 | **6.51** | **4.48** | **7.58** |
| 1605 | ?3458 | IVGN-novel-miR_3458 | **0.10** | **1.27** | **1.68** |
| 1864 | ?3459 | IVGN-novel-miR_3459 | **1.26** | **0.29** | **0.39** |
| 1863 | ?3460 | IVGN-novel-miR_3460 | **1.05** | **1.18** | **1.37** |
| 1862 | ?3461 | IVGN-novel-miR_3461 | **1.00** | **0.33** | **0.41** |
| 1861 | ?3462 | IVGN-novel-miR_3462 | **1.67** | **5.39** | **7.81** |
| 2120 | ?3463 | IVGN-novel-miR_3463 | **62.32** | **65.43** | **65.04** |
| 2118 | ?3465 | IVGN-novel-miR_3465 | **3.47** | **3.44** | **5.36** |
| 2117 | ?3466 | IVGN-novel-miR_3466 | **3.46** | **5.19** | **6.28** |
| 2376 | ?3467 | IVGN-novel-miR_3467 | **0.81** | **1.68** | **1.61** |
| 2375 | ?3468 | IVGN-novel-miR_3468 | **13.34** | **44.82** | **41.93** |
| 2374 | ?3469 | IVGN-novel-miR_3469 | **4.76** | **4.19** | **3.76** |
| 2373 | ?3470 | IVGN-novel-miR_3470 | **18.19** | **103.87** | **90.25** |
| 2632 | ?3471 | IVGN-novel-miR_3471 | **27.96** | **34.59** | **22.04** |
| 2631 | ?3472 | IVGN-novel-miR_3472 | **12.44** | **38.72** | **20.66** |
| 2629 | ?3474 | IVGN-novel-miR_3474 | **1.00** | **0.70** | **0.52** |
| 2888 | ?3475 | IVGN-novel-miR_3475 | **32.78** | **27.43** | **6.49** |
| 2887 | ?3476 | IVGN-novel-miR_3476 | **0.75** | **0.19** | **0.26** |
| 2886 | ?3477 | IVGN-novel-miR_3477 | **6.42** | **2.88** | **2.67** |
| 2885 | ?3478 | IVGN-novel-miR_3478 | **0.52** | **0.07** | **0.08** |
| 88 | ?3479 | IVGN-novel-miR_3479 | **1.36** | **0.93** | **0.60** |
| 87 | ?3480 | IVGN-novel-miR_3480 | **29.65** | **20.95** | **19.30** |
| 86 | ?3481 | IVGN-novel-miR_3481 | **0.86** | **0.57** | **0.46** |
| 85 | ?3482 | IVGN-novel-miR_3482 | **1.05** | **0.49** | **0.36** |
| 344 | ?3483 | IVGN-novel-miR_3483 | **0.44** | **0.04** | **0.08** |
| 343 | ?3484 | IVGN-novel-miR_3484 | **1.38** | **0.47** | **0.62** |
| 341 | ?3486 | IVGN-novel-miR_3486 | **1.01** | **1.62** | **1.36** |
| 600 | ?3487 | IVGN-novel-miR_3487 | **4.55** | **2.18** | **7.52** |
| 599 | ?3488 | IVGN-novel-miR_3488 | **20.87** | **57.79** | **61.42** |
| 598 | ?3489 | IVGN-novel-miR_3489 | **2.99** | **1.53** | **7.55** |
| 597 | ?3490 | IVGN-novel-miR_3490 | **0.43** | **0.30** | **0.96** |
| 856 | ?3491 | IVGN-novel-miR_3491 | **0.82** | **0.59** | **0.92** |
| 855 | ?3492 | IVGN-novel-miR_3492 | **0.56** | **0.43** | **0.74** |
| 854 | ?3493 | IVGN-novel-miR_3493 | **0.42** | **0.15** | **0.27** |
| 853 | ?3494 | IVGN-novel-miR_3494 | **0.54** | **0.20** | **0.34** |
| 1112 | ?3495 | IVGN-novel-miR_3495 | **2.23** | **0.87** | **1.85** |
| 1111 | ?3496 | IVGN-novel-miR_3496 | **6.96** | **0.37** | **0.22** |
| 1110 | ?3497 | IVGN-novel-miR_3497 | **66.45** | **3.98** | **18.43** |
| 1109 | ?3498 | IVGN-novel-miR_3498 | **1.42** | **1.27** | **2.08** |
| 1368 | ?3499 | IVGN-novel-miR_3499 | **8.55** | **3.87** | **8.28** |
| 1367 | ?3500 | IVGN-novel-miR_3500 | **0.68** | **0.43** | **0.42** |
| 1366 | ?3501 | IVGN-novel-miR_3501 | **0.35** | **0.32** | **0.41** |
| 1365 | ?3502 | IVGN-novel-miR_3502 | **26.75** | **91.99** | **110.94** |
| 1624 | ?3503 | IVGN-novel-miR_3503 | **0.86** | **0.25** | **0.32** |
| 1623 | ?3504 | IVGN-novel-miR_3504 | **120.89** | **195.06** | **193.01** |
| 1622 | ?3505 | IVGN-novel-miR_3505 | **1.68** | **1.24** | **1.93** |
| 1621 | ?3506 | IVGN-novel-miR_3506 | **1.01** | **1.05** | **1.33** |
| 1880 | ?3507 | IVGN-novel-miR_3507 | **9.75** | **16.57** | **20.04** |
| 1879 | ?3508 | IVGN-novel-miR_3508 | **1.02** | **0.60** | **0.55** |
| 1878 | ?3509 | IVGN-novel-miR_3509 | **13.70** | **10.56** | **14.27** |
| 1877 | ?3510 | IVGN-novel-miR_3510 | **0.51** | **0.36** | **0.26** |
| 2136 | ?3511 | IVGN-novel-miR_3511 | **54.56** | **91.56** | **121.32** |
| 2135 | ?3512 | IVGN-novel-miR_3512 | **1.19** | **0.67** | **0.60** |
| 2134 | ?3513 | IVGN-novel-miR_3513 | **25.26** | **14.73** | **19.03** |
| 2133 | ?3514 | IVGN-novel-miR_3514 | **3.29** | **6.17** | **5.69** |
| 2392 | ?3515 | IVGN-novel-miR_3515 | **209.44** | **225.07** | **212.74** |
| 2391 | ?3516 | IVGN-novel-miR_3516 | **73.07** | **187.04** | **181.60** |
| 2390 | ?3517 | IVGN-novel-miR_3517 | **2.81** | **0.55** | **0.41** |
| 2389 | ?3518 | IVGN-novel-miR_3518 | **30.06** | **14.98** | **16.72** |
| 2647 | ?3520 | IVGN-novel-miR_3520 | **0.66** | **0.21** | **0.20** |
| 2646 | ?3521 | IVGN-novel-miR_3521 | **18.75** | **4.71** | **3.34** |
| 2645 | ?3522 | IVGN-novel-miR_3522 | **4.55** | **5.75** | **2.78** |
| 2904 | ?3523 | IVGN-novel-miR_3523 | **7.95** | **9.48** | **2.27** |
| 2903 | ?3524 | IVGN-novel-miR_3524 | **0.94** | **0.66** | **0.63** |
| 2902 | ?3525 | IVGN-novel-miR_3525 | **4.52** | **2.26** | **0.96** |
| 2901 | ?3526 | IVGN-novel-miR_3526 | **7.03** | **4.66** | **1.38** |
| 76 | ?3527 | IVGN-novel-miR_3527 | **7.44** | **4.31** | **5.19** |
| 332 | ?3531 | IVGN-novel-miR_3531 | **2.08** | **7.95** | **8.56** |
| 330 | ?3533 | IVGN-novel-miR_3533 | **0.73** | **0.43** | **0.39** |
| 329 | ?3534 | IVGN-novel-miR_3534 | **0.91** | **1.04** | **1.29** |
| 588 | ?3535 | IVGN-novel-miR_3535 | **3.90** | **2.79** | **3.52** |
| 587 | ?3536 | IVGN-novel-miR_3536 | **33.45** | **16.39** | **19.30** |
| 586 | ?3537 | IVGN-novel-miR_3537 | **75.51** | **118.36** | **180.72** |
| 585 | ?3538 | IVGN-novel-miR_3538 | **4.00** | **14.44** | **16.05** |
| 844 | ?3539 | IVGN-novel-miR_3539 | **0.97** | **0.66** | **0.63** |
| 843 | ?3540 | IVGN-novel-miR_3540 | **0.62** | **0.23** | **0.24** |
| 842 | ?3541 | IVGN-novel-miR_3541 | **1.75** | **0.57** | **1.53** |
| 841 | ?3542 | IVGN-novel-miR_3542 | **1.87** | **0.75** | **1.96** |
| 1100 | ?3543 | IVGN-novel-miR_3543 | **2.02** | **0.41** | **0.43** |
| 1099 | ?3544 | IVGN-novel-miR_3544 | **48.32** | **16.94** | **26.13** |
| 1098 | ?3545 | IVGN-novel-miR_3545 | **0.43** | **0.24** | **0.42** |
| 1097 | ?3546 | IVGN-novel-miR_3546 | **9.31** | **15.07** | **17.55** |
| 1355 | ?3548 | IVGN-novel-miR_3548 | **1.04** | **0.48** | **0.54** |
| 1354 | ?3549 | IVGN-novel-miR_3549 | **5.55** | **37.76** | **50.38** |
| 1353 | ?3550 | IVGN-novel-miR_3550 | **0.95** | **0.23** | **0.31** |
| 1612 | ?3551 | IVGN-novel-miR_3551 | **0.70** | **0.53** | **0.43** |
| 1611 | ?3552 | IVGN-novel-miR_3552 | **5.48** | **2.26** | **3.65** |
| 1865 | ?3558 | IVGN-novel-miR_3558 | **0.66** | **0.11** | **0.31** |
| 2124 | ?3559 | IVGN-novel-miR_3559 | **99.69** | **109.43** | **134.95** |
| 2123 | ?3560 | IVGN-novel-miR_3560 | **0.58** | **0.78** | **1.08** |
| 2121 | ?3562 | IVGN-novel-miR_3562 | **0.61** | **0.52** | **0.31** |
| 2380 | ?3563 | IVGN-novel-miR_3563 | **7.28** | **1.64** | **1.48** |
| 2379 | ?3564 | IVGN-novel-miR_3564 | **9.18** | **36.79** | **32.37** |
| 2378 | ?3565 | IVGN-novel-miR_3565 | **1.14** | **3.48** | **3.49** |
| 2635 | ?3568 | IVGN-novel-miR_3568 | **4.22** | **2.40** | **1.71** |
| 2634 | ?3569 | IVGN-novel-miR_3569 | **0.60** | **0.07** | **0.09** |
| 2633 | ?3570 | IVGN-novel-miR_3570 | **5.26** | **7.02** | **4.77** |
| 2891 | ?3572 | IVGN-novel-miR_3572 | **0.65** | **0.12** | **0.05** |
| 2890 | ?3573 | IVGN-novel-miR_3573 | **29.44** | **20.69** | **4.34** |
| 2889 | ?3574 | IVGN-novel-miR_3574 | **1.17** | **0.62** | **0.36** |
| 92 | ?3575 | IVGN-novel-miR_3575 | **0.49** | **0.06** | **0.05** |
| 91 | ?3576 | IVGN-novel-miR_3576 | **0.04** | **1.90** | **0.11** |
| 90 | ?3577 | IVGN-novel-miR_3577 | **21.79** | **14.07** | **16.91** |
| 89 | ?3578 | IVGN-novel-miR_3578 | **0.43** | **0.12** | **0.14** |
| 348 | ?3579 | IVGN-novel-miR_3579 | **9.74** | **9.61** | **11.73** |
| 347 | ?3580 | IVGN-novel-miR_3580 | **0.29** | **0.34** | **0.34** |
| 346 | ?3581 | IVGN-novel-miR_3581 | **0.56** | **0.57** | **0.53** |
| 345 | ?3582 | IVGN-novel-miR_3582 | **1.06** | **0.69** | **0.72** |
| 601 | ?3586 | IVGN-novel-miR_3586 | **1.04** | **0.32** | **0.55** |
| 860 | ?3587 | IVGN-novel-miR_3587 | **22.04** | **13.19** | **18.38** |
| 857 | ?3590 | IVGN-novel-miR_3590 | **17.40** | **41.82** | **55.14** |
| 1116 | ?3591 | IVGN-novel-miR_3591 | **10.85** | **22.92** | **20.99** |
| 1114 | ?3593 | IVGN-novel-miR_3593 | **4.10** | **1.85** | **4.39** |
| 1113 | ?3594 | IVGN-novel-miR_3594 | **10.93** | **6.22** | **7.81** |
| 1372 | ?3595 | IVGN-novel-miR_3595 | **13.95** | **8.58** | **9.80** |
| 1628 | ?3599 | IVGN-novel-miR_3599 | **9.82** | **5.75** | **6.14** |
| 1626 | ?3601 | IVGN-novel-miR_3601 | **0.44** | **0.03** | **0.05** |
| 1625 | ?3602 | IVGN-novel-miR_3602 | **2.46** | **0.16** | **0.19** |
| 1884 | ?3603 | IVGN-novel-miR_3603 | **2.73** | **0.47** | **0.59** |
| 1882 | ?3605 | IVGN-novel-miR_3605 | **0.66** | **0.10** | **0.19** |
| 1881 | ?3606 | IVGN-novel-miR_3606 | **53.13** | **45.83** | **46.01** |
| 2140 | ?3607 | IVGN-novel-miR_3607 | **0.74** | **0.49** | **0.40** |
| 2394 | ?3613 | IVGN-novel-miR_3613 | **1.57** | **1.16** | **1.01** |
| 2393 | ?3614 | IVGN-novel-miR_3614 | **5.05** | **3.07** | **2.64** |
| 2652 | ?3615 | IVGN-novel-miR_3615 | **5.84** | **1.38** | **1.00** |
| 2651 | ?3616 | IVGN-novel-miR_3616 | **0.51** | **0.68** | **0.50** |
| 2650 | ?3617 | IVGN-novel-miR_3617 | **3.61** | **1.02** | **0.72** |
| 2649 | ?3618 | IVGN-novel-miR_3618 | **4.64** | **2.09** | **1.30** |
| 2908 | ?3619 | IVGN-novel-miR_3619 | **1.50** | **0.68** | **0.51** |
| 2906 | ?3621 | IVGN-novel-miR_3621 | **0.63** | **0.15** | **0.38** |
| 2905 | ?3622 | IVGN-novel-miR_3622 | **1.03** | **0.90** | **0.59** |
| 80 | ?3623 | IVGN-novel-miR_3623 | **31.68** | **39.85** | **23.30** |
| 79 | ?3624 | IVGN-novel-miR_3624 | **0.88** | **0.49** | **0.30** |
| 78 | ?3625 | IVGN-novel-miR_3625 | **37.45** | **90.06** | **79.96** |
| 77 | ?3626 | IVGN-novel-miR_3626 | **10.09** | **16.15** | **15.46** |
| 336 | ?3627 | IVGN-novel-miR_3627 | **0.28** | **3.00** | **2.29** |
| 335 | ?3628 | IVGN-novel-miR_3628 | **3.02** | **3.60** | **4.30** |
| 334 | ?3629 | IVGN-novel-miR_3629 | **7.81** | **9.04** | **6.61** |
| 333 | ?3630 | IVGN-novel-miR_3630 | **1.03** | **0.70** | **0.67** |
| 592 | ?3631 | IVGN-novel-miR_3631 | **0.90** | **0.64** | **0.43** |
| 591 | ?3632 | IVGN-novel-miR_3632 | **1.10** | **0.65** | **0.80** |
| 590 | ?3633 | IVGN-novel-miR_3633 | **0.97** | **0.51** | **0.50** |
| 589 | ?3634 | IVGN-novel-miR_3634 | **1.80** | **1.00** | **0.94** |
| 848 | ?3635 | IVGN-novel-miR_3635 | **0.42** | **0.26** | **0.21** |
| 847 | ?3636 | IVGN-novel-miR_3636 | **1.08** | **0.52** | **0.44** |
| 846 | ?3637 | IVGN-novel-miR_3637 | **1.57** | **0.84** | **0.87** |
| 845 | ?3638 | IVGN-novel-miR_3638 | **1.37** | **0.95** | **0.78** |
| 1104 | ?3639 | IVGN-novel-miR_3639 | **0.50** | **0.22** | **0.14** |
| 1103 | ?3640 | IVGN-novel-miR_3640 | **1.86** | **1.05** | **1.09** |
| 1360 | ?3643 | IVGN-novel-miR_3643 | **0.16** | **0.68** | **1.24** |
| 1359 | ?3644 | IVGN-novel-miR_3644 | **1.32** | **0.75** | **0.69** |
| 1357 | ?3646 | IVGN-novel-miR_3646 | **3.25** | **0.77** | **1.01** |
| 1616 | ?3647 | IVGN-novel-miR_3647 | **5.43** | **0.58** | **0.98** |
| 1613 | ?3650 | IVGN-novel-miR_3650 | **131.28** | **32.52** | **49.61** |
| 1872 | ?3651 | IVGN-novel-miR_3651 | **1.13** | **0.51** | **0.38** |
| 1871 | ?3652 | IVGN-novel-miR_3652 | **3.31** | **1.88** | **1.65** |
| 1870 | ?3653 | IVGN-novel-miR_3653 | **0.45** | **0.18** | **0.11** |
| 1869 | ?3654 | IVGN-novel-miR_3654 | **0.46** | **0.20** | **0.21** |
| 2128 | ?3655 | IVGN-novel-miR_3655 | **1.35** | **0.62** | **0.78** |
| 2127 | ?3656 | IVGN-novel-miR_3656 | **63.83** | **109.12** | **144.98** |
| 2126 | ?3657 | IVGN-novel-miR_3657 | **52.94** | **103.02** | **136.53** |
| 2125 | ?3658 | IVGN-novel-miR_3658 | **34.78** | **81.51** | **109.11** |
| 2384 | ?3659 | IVGN-novel-miR_3659 | **0.88** | **0.25** | **0.32** |
| 2383 | ?3660 | IVGN-novel-miR_3660 | **4.83** | **1.04** | **0.98** |
| 2382 | ?3661 | IVGN-novel-miR_3661 | **9.64** | **3.80** | **2.78** |
| 2381 | ?3662 | IVGN-novel-miR_3662 | **3.93** | **0.79** | **0.94** |
| 2640 | ?3663 | IVGN-novel-miR_3663 | **6.74** | **2.49** | **1.54** |
| 2639 | ?3664 | IVGN-novel-miR_3664 | **4.44** | **1.72** | **1.21** |
| 2638 | ?3665 | IVGN-novel-miR_3665 | **2.74** | **1.09** | **0.79** |
| 2637 | ?3666 | IVGN-novel-miR_3666 | **163.49** | **23.59** | **18.09** |
| 2896 | ?3667 | IVGN-novel-miR_3667 | **0.61** | **0.16** | **0.15** |
| 2895 | ?3668 | IVGN-novel-miR_3668 | **12.81** | **5.32** | **2.18** |
| 2894 | ?3669 | IVGN-novel-miR_3669 | **12.50** | **4.46** | **2.17** |
| 2893 | ?3670 | IVGN-novel-miR_3670 | **7.52** | **2.66** | **1.25** |
| 95 | ?3672 | IVGN-novel-miR_3672 | **0.47** | **0.51** | **0.48** |
| 94 | ?3673 | IVGN-novel-miR_3673 | **56.65** | **204.99** | **183.05** |
| 93 | ?3674 | IVGN-novel-miR_3674 | **1.61** | **1.20** | **0.97** |
| 352 | ?3675 | IVGN-novel-miR_3675 | **0.59** | **0.03** | **0.18** |
| 351 | ?3676 | IVGN-novel-miR_3676 | **1.18** | **1.05** | **1.68** |
| 350 | ?3677 | IVGN-novel-miR_3677 | **1.17** | **0.85** | **0.78** |
| 349 | ?3678 | IVGN-novel-miR_3678 | **0.52** | **0.18** | **0.10** |
| 606 | ?3681 | IVGN-novel-miR_3681 | **7.41** | **5.03** | **8.90** |
| 605 | ?3682 | IVGN-novel-miR_3682 | **0.75** | **0.41** | **0.44** |
| 862 | ?3685 | IVGN-novel-miR_3685 | **1.44** | **0.89** | **0.71** |
| 1373 | ?3694 | IVGN-novel-miR_3686 | **0.69** | **0.08** | **0.17** |
| 861 | ?3686 | IVGN-novel-miR_3686 | **8.17** | **5.80** | **10.32** |
| 1119 | ?3688 | IVGN-novel-miR_3688 | **2.63** | **1.95** | **1.84** |
| 1118 | ?3689 | IVGN-novel-miR_3689 | **4.43** | **4.41** | **6.67** |
| 1376 | ?3691 | IVGN-novel-miR_3691 | **12.47** | **5.68** | **5.58** |
| 1374 | ?3693 | IVGN-novel-miR_3693 | **0.66** | **0.27** | **0.44** |
| 1630 | ?3697 | IVGN-novel-miR_3697 | **0.55** | **0.22** | **0.39** |
| 1629 | ?3698 | IVGN-novel-miR_3698 | **0.42** | **0.16** | **0.08** |
| 1888 | ?3699 | IVGN-novel-miR_3699 | **3.60** | **2.32** | **2.01** |
| 1887 | ?3700 | IVGN-novel-miR_3700 | **8.17** | **49.59** | **46.40** |
| 1886 | ?3701 | IVGN-novel-miR_3701 | **2.17** | **7.20** | **8.13** |
| 1885 | ?3702 | IVGN-novel-miR_3702 | **16.19** | **5.63** | **7.35** |
| 514 | Positive control-2 (N) | | **45.58** | **4.69** | **5.08** |
| 258 | Positive control-2 (N) | | **54.20** | **8.02** | **5.66** |
| 770 | Positive control-2 (N) | | **37.68** | **5.79** | **7.52** |
| 513 | Positive control-1 (G) | | **56.83** | **9.71** | **8.37** |
| 2 | Positive control-2 (N) | | **65.15** | **13.61** | **8.48** |
| 257 | Positive control-1 (G) | | **60.78** | **14.19** | **8.91** |
| 769 | Positive control-1 (G) | | **39.73** | **9.09** | **9.06** |
| 1 | Positive control-1 (G) | | **56.29** | **16.34** | **10.61** |
| 259 | Positive control-3 (B) | | **55.25** | **24.51** | **18.92** |
| 515 | Positive control-3 (B) | | **46.81** | **16.98** | **20.12** |
| 3 | Positive control-3 (B) | | **71.54** | **29.94** | **20.28** |
| 771 | Positive control-3 (B) | | **31.91** | **13.92** | **20.65** |
