# Supplementary material for: Microvesicles secreted by macrophages shuttle invasion-potentiating microRNAs into breast cancer cells
Source: Mol Cancer. 2011 Sep 22;10:117. doi: 10.1186/1476-4598-10-117 (PMC3190352; doi:10.1186/1476-4598-10-117)
Supplement: Additional file 6 — Figure S5. Exosomes derived from IL-4 activated macrophages treated by RNase plus triton X-100 had decreased invasion potentiate. Exosomes secreted from IL-4 activated macrophages or unactivated macrophages were treated by RNase with or without triton X-100. SKBR3 breast cancer cells were then incubated with those exosomes. Invasion assays were performed. Data are presented as the number of invading cells per field (A) and representative images of invading cells were showed in (B). ** p < 0.01. (Un-Mac, unactivated macrophages; IL4-Mac, IL-4-activated macrophages). [file 1476-4598-10-117-S6.PDF]

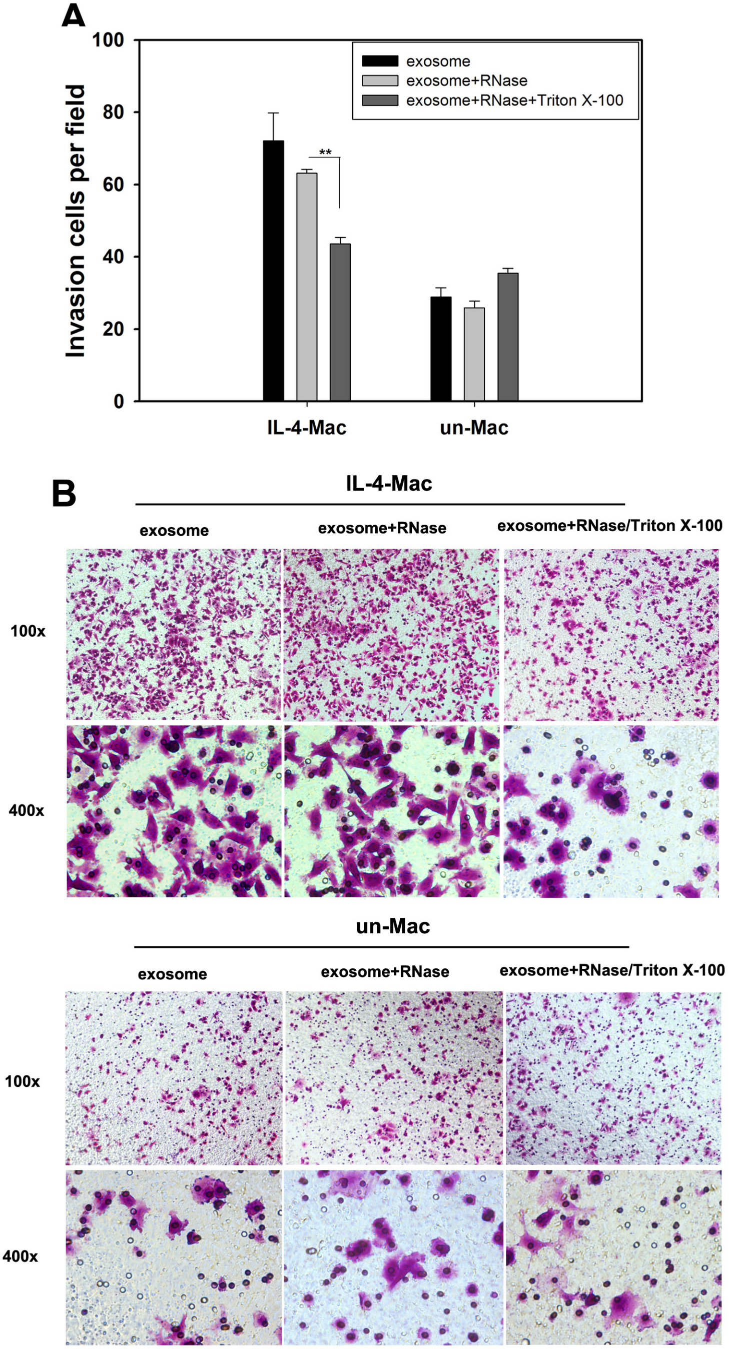
A
Invasion cells per field
0
20
40
60
80
100
exosome
exosome+RNase
exosome+RNase+Triton X-100
**
IL-4-Mac
un-Mac
B
IL-4-Mac
exosome
exosome+RNase
exosome+RNase/Triton X-100
100x
400x
un-Mac
exosome
exosome+RNase
exosome+RNase/Triton X-100
100x
400x
